# Efficient electrocatalytic nitrogen reduction to ammonia with aqueous silver nanodots

**DOI:** 10.1038/s42004-021-00449-7

**Published:** 2021-01-29

**Authors:** Wenyi Li, Ke Li, Yixing Ye, Shengbo Zhang, Yanyan Liu, Guozhong Wang, Changhao Liang, Haimin Zhang, Huijun Zhao

**Affiliations:** 1 Key Laboratory of Materials Physics, Centre for Environmental and Energy Nanomaterials, Anhui Key Laboratory of Nanomaterials and Nanotechnology, CAS Center for Excellence in Nanoscience, Institute of Solid State Physics, HFIPS, Chinese Academy of Sciences, Hefei, 230031 China; 2 University of Science and Technology of China, Hefei, 230026 China; 3 Centre for Clean Environment and Energy, Griffith University, Gold Coast Campus, Griffith, QLD Australia

**Keywords:** Quantum dots, Electrocatalysis, Electrocatalysis, Sustainability

## Abstract

The electrocatalytic nitrogen (N_2_) reduction reaction (NRR) relies on the development of highly efficient electrocatalysts and electrocatalysis systems. Herein, we report a non-loading electrocatalysis system, where the electrocatalysts are dispersed in aqueous solution rather than loading them on electrode substrates. The system consists of aqueous Ag nanodots (AgNDs) as the catalyst and metallic titanium (Ti) mesh as the current collector for electrocatalytic NRR. The as-synthesized AgNDs, homogeneously dispersed in 0.1 M Na_2_SO_4_ solution (pH = 10.5), can achieve an NH_3_ yield rate of 600.4 ± 23.0 μg h^−1^ mg_Ag_^−1^ with a faradaic efficiency (FE) of 10.1 ± 0.7% at −0.25 V (vs. RHE). The FE can be further improved to be 20.1 ± 0.9% at the same potential by using Ti mesh modified with oxygen vacancy-rich TiO_2_ nanosheets as the current collector. Utilizing the aqueous AgNDs catalyst, a Ti plate based two-electrode configured flow-type electrochemical reactor was developed to achieve an NH_3_ yield rate of 804.5 ± 30.6 μg h^−1^ mg_Ag_^−1^ with a FE of 8.2 ± 0.5% at a voltage of −1.8 V. The designed non-loading electrocatalysis system takes full advantage of the AgNDs’ active sites for N_2_ adsorption and activation, following an alternative hydrogenation mechanism revealed by theoretical calculations.

## Abstract (web summary)

Nano-sized catalysts are useful for electrocatalytic nitrogen reduction, but loading the particles on electrode substrates can limit catalytic performance. Here, the authors report nitrogen to ammonia electroreduction mediated by silver nanodots dispersed in an aqueous solution.

## Introduction

The fixation of nitrogen (N_2_) to ammonia (NH_3_) at room temperature and atmospheric pressure is an attractive but greatly challenging topic^1–3^. Currently, the over century-old Haber-Bosch process is still predominant for industrial-scale NH_3_ production. However, this process needs to consume tremendous energy and natural gas, and concurrently release large amount of CO_2_^4–6^, very adverse for energy and environmental sustainability. In recent years, ambient electrocatalytic reduction of N_2_ to NH_3_ has attracted increasing attention because of its mild operation conditions, water as the hydrogen source and no CO_2_ emission^5–9^. However, the electrocatalytic N_2_ reduction technique is still far away from the practical application because the developed nitrogen reduction reaction (NRR) electrocatalysts possess low NH_3_ yield and current efficiency, mainly due to extremely high stability of N_2_ and competitive hydrogen evolution reaction (HER)^6–11^. Therefore, development of high-efficiency NRR electrocatalysts and catalysis system is highly desirable for future industrial NH_3_ production at ambient conditions.

It is well known that reducing the size of electrocatalysts to very small particles has been regarded as an efficient approach to achieve more catalytic active sites for high-efficiency electrocatalysis^12–16^. Owing to unique physical and chemical properties, carbon- and metal-based nanodots (the sizes < 10 nm) have been widely applied to photovoltaic devices, environmental fluorescence detection, photocatalysis and electrocatalysis^17–19^. Recently, transition metal oxides and carbides nanodots supported on carbon substrates have been fabricated for the NRR, exhibiting good electrocatalytic activities^20–25^. Although an individual nanodot can provide abundant catalytic active sites (e.g., facet, edge, corner, surface-rich O/N functional sites) during electrocatalytic NRR, the nanodots’ NRR performance is still very low (NH_3_ yield rate < ~25 μg h^−1^ mg^−1^ and faradaic efficiency < ~10%) in the reported literatures^20–25^. This could be primarily attributed to two factors: (i) a limited loading amount of nanodots catalysts on carbon substrates means limited catalytic active sites utilization for NRR; (ii) the loading approach of nanodots catalysts onto the electrode supports readily results in their aggregation during NRR, thus greatly decreasing the exposed catalytic active sites. On the basis of the reported works, some fabricated nanodots can be highly dispersed into aqueous solution. This provides us an opportunity to develop a non-loading electrocatalysis system, capable of employing the nanodots catalysts in their highly dispersed form in aqueous solution, which may be an effective means to sufficiently utilize the exposed catalytic active sites provided by nanodots for high-efficiency NRR. Recently, Ag nanosheets, nanoporous film, triangular nanoplates, Ag-Au, Ag-Cu alloying materials and Ag single-atom catalysts, have been developed for electrocatalytic NRR, displaying high NRR activities^26–31^. It can be envisaged that small-sized Ag nanodots with abundant catalytic active sites (e.g., facet, edge, corner etc.)^12,14,16^ may be more promising candidate for electrocatalytic NRR. However, how to effectively utilize highly dispersed Ag nanodots catalyst in aqueous solution is an important issue that needs to be solved.

Herein, we report the laser-ablation technique to fabricate highly dispersed Ag nanodots (AgNDs) in aqueous solution under Ar atmosphere^32^. The as-synthesized AgNDs with an average nanodot size of ~2.3 nm are subsequently employed to a non-loading electrocatalysis system, composed of metallic titanium (Ti) mesh as the current collector and AgNDs as the electrocatalyst dispersed in 0.1 M Na_2_SO_4_ solution (pH = 10.5) for nitrogen reduction reaction (NRR) to NH_3_. The aqueous AgNDs with abundant catalytic active sites can effectively chemisorb the dissolved N_2_ molecules in electrolyte, then transfer to the Ti mesh current collector under stirring to accept the H^+^/e^−^ attack for NH_3_ formation and concurrently regenerate the AgNDs (Supplementary Fig. 1a). Comparatively, in a conventional catalyst-loading electrocatalysis process (Supplementary Fig. 1b), the as-synthesized AgNDs are coated on the electrode substrate (e.g., commercial carbon cloth) with limited loading amount as the cathode, which could arouse an aggregation of AgNDs during NRR and concurrently suffer from the binder’s adverse influence (for the electrode preparation)^33,34^. These factors may significantly decrease the NRR performance of AgNDs catalyst. As a result, utilizing the developed non-loading electrocatalysis system, a large NH_3_ yield rate of 600.4 ± 23.0 μg h^−1^ mg_Ag_^−1^ with a faradaic efficiency (FE) of 10.1 ± 0.7% can be achieved at −0.25 V (vs. RHE) in 0.1 M Na_2_SO_4_ electrolyte. The NH_3_ yield and FE of this system can be further improved by oxygen vacancies-rich TiO_2_ nanosheets modified Ti mesh as the current collector, surpassing most of recently reported nanodots and other electrocatalysts for aqueous NRR (Supplementary Table 1). A two-electrode configured flow-type electrochemical reactor using aqueous AgNDs catalyst is therefore developed to obtain an NH_3_ yield rate of 804.5 ± 30.6 μg h^−1^ mg_Ag_^−1^ with a FE of 8.2 ± 0.5% at a voltage of −1.8 V. The NRR active mechanism of AgNDs is revealed by our theoretical calculations results.

## Results

### Synthesis and characterization of AgNDs

**Fig. 1:**
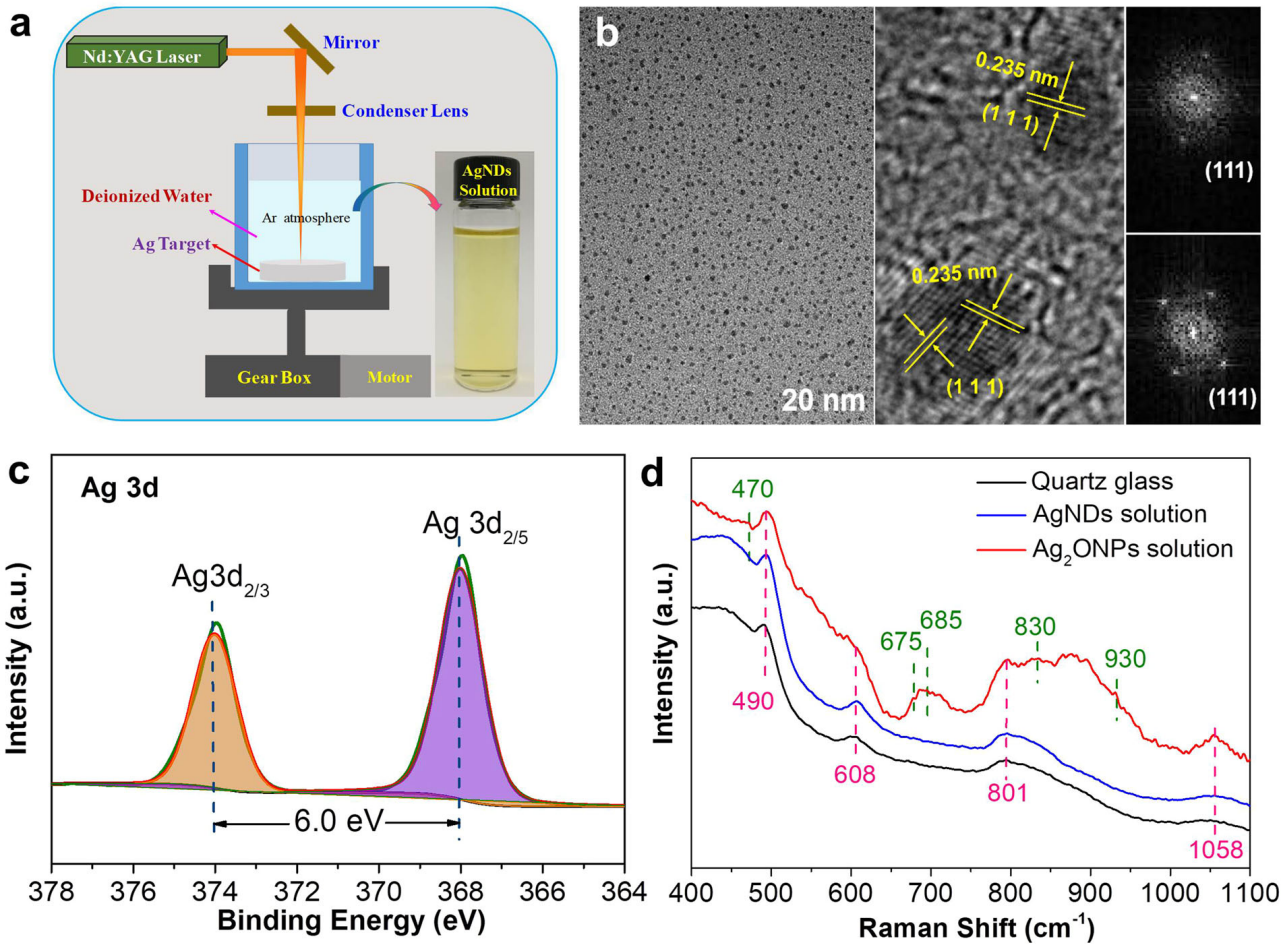
Characteristics of AgNDs. **a** Schematic illustration of the fabrication process of Ag nanodots (AgNDs) solution (inset of optical photograph of AgNDs solution) by the laser-ablation technique. **b** TEM and HRTEM images (inset of corresponding FFT patterns) of AgNDs. **c** High-resolution Ag 3d XPS spectrum of AgNDs. **d** Raman spectra of quartz glass substrate, AgNDs and Ag_2_ ONPs reference.

In this work, we employed the laser-ablation technique to fabricate the uniformly dispersed Ag nanodots in deionized water under Ar atmosphere (Fig. 1a). Figure 1b and Supplementary Fig. 2 show the TEM images of the as-synthesized AgNDs, exhibiting very homogeneous nanodot morphology with an average nanodot size of ~2.3 nm (inset in Supplementary Fig. 2). The high-resolution TEM (HRTEM) image of the AgNDs (Fig. 1b) shows the lattice spacing of 0.235 nm, corresponding to the (111) plane of fcc-phase metallic Ag. The corresponding fast Fourier transform (FFT) patterns further confirm this (inset in Fig. 1b). The above results demonstrate that the laser-ablation fabricated AgNDs exhibit highly exposed (111) planes, possibly beneficial for electrocatalysis^16,27^. Supplementary Fig. 3a presents the surface survey XPS spectrum of AgNDs, indicating the presence of Ag and O elements. The high-resolution O1s XPS spectrum (Supplementary Fig. 3b) shows that the only peak located at ~532.4 eV is resulted from the surface adsorbed OH^−^/H_2_O^35^. In this work, the Zeta potential of AgNDs in aqueous solution was measured to be −41.7 mV, ascribed to the presence of OH^−^ groups on the surface^36^. This is the reason why the fabricated AgNDs can highly disperse in aqueous solution. The pH value of AgNDs aqueous solution was measured to be 9.5, supportive of the Zeta potential result. Figure 1c displays the high-resolution Ag 3d XPS spectrum of AgNDs. The peaks concentrated at ~368.1 and ~374.1 eV can be associated with Ag 3d5/2 and Ag 3d3/2, suggesting the existence of metallic Ag phase in AgNDs^37,38^. This can be further confirmed by the Raman spectra characterization. As shown in Fig. 1d, the peaks concentrated at 490, 608, 801 and 1058 cm^−1^ for all samples can be indexed to four-membered SiO rings, three-membered SiO rings, Si-O-Si and Si-OH originated from quartz glass substrate, respectively^39^. Comparatively, the AgNDs sample exhibits the similar Raman peaks as quartz glass substrate, meaning that no oxidation-state Ag is existent (e.g., Ag_2_O nanoparticles (Ag_2_ONPs), typical Ag-O peaks at 470, 675, 685, 830 cm^−1^ and O-O peak at 930 cm^−1^ can be confirmed in our Ag_2_ONPs refs. ^35,40,41^). The above results demonstrate that highly dispersed Ag nanodots in aqueous solution are successfully achieved by the laser-ablation technique, possibly providing abundant active sites for high-efficiency electrocatalysis^42^.

### NRR performance evaluation

**Fig. 2:**
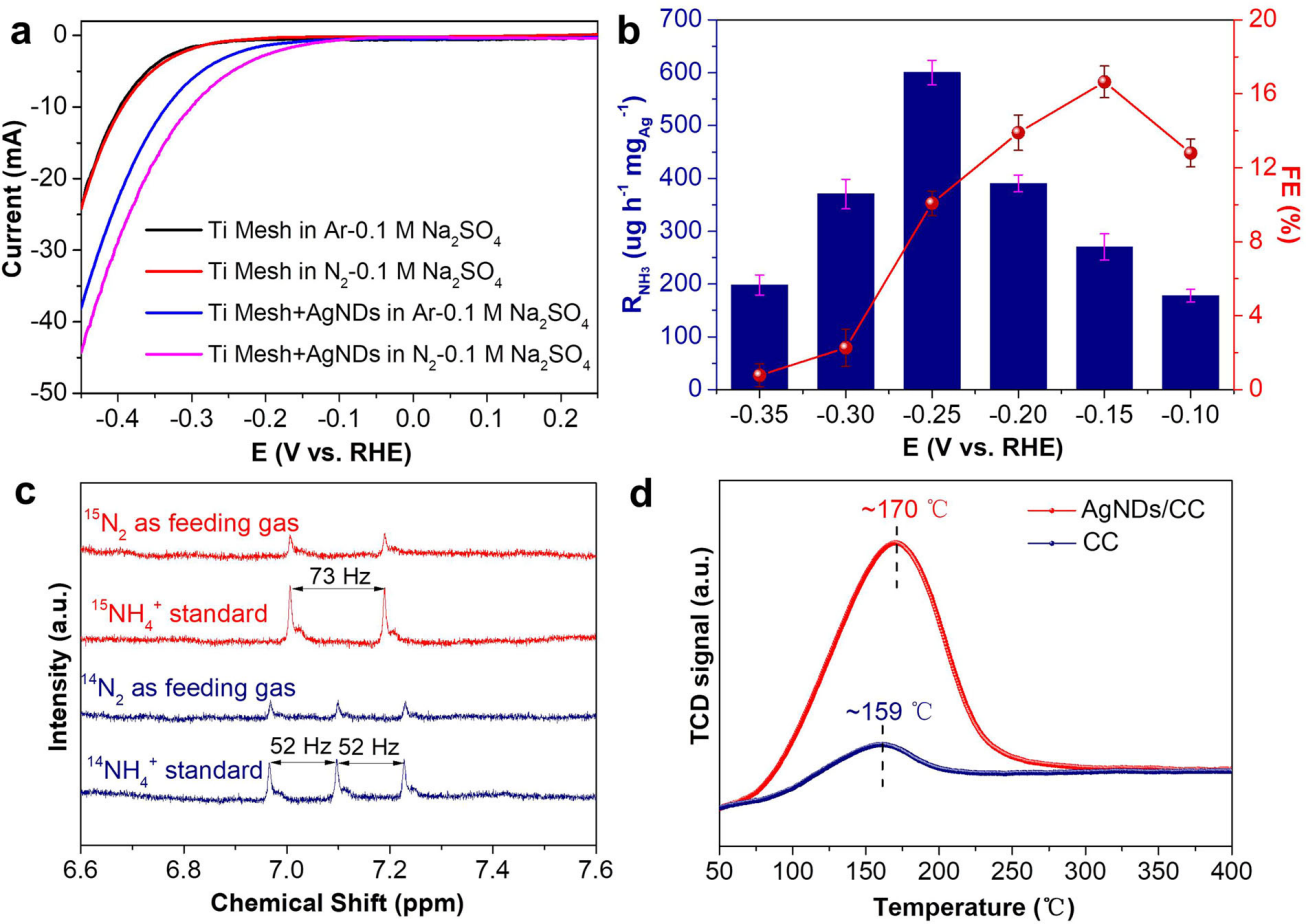
NRR evaluation of AgNDs in the non-loading electrocatalysis system. **a** LSV curves of Ti mesh with and without AgNDs incorporation in Ar- and N_2_-saturated 0.1 M Na_2_SO_4_ solutions (pH = 10.5). **b** The dependence of NH_3_ yield rate and faradaic efficiency of AgNDs catalyst on the applied potential. The error bars correspond to the standard deviations of three independent measurements. **c** ^1^H NMR spectra of the samples obtained using ^15^N_2_ and ^14^N_2_ as the feeding gases and standard ^15^NH_4_^+^ and ^14^NH_4_^+^ samples. **d** N_2_-TPD curves of CC and AgNDs/CC.

Recently, transition metal oxides and carbides nanodots such as CoO, CuO, NiO, ZnO and MoC have been investigated as the electrocatalysts for NRR, exhibiting good catalytic activities^20–23,25^. However, these obtained nanodots are exclusively anchored on carbon substrates in the fabrication process, meaning that the nanodots loading amount could be very limited, thus resulting in low electrocatalytic NRR performance^20–25,43,44^. In this work, we utilized the developed non-loading electrocatalysis system to evaluate the NRR activity of AgNDs uniformly dispersed in 0.1 M Na_2_SO_4_ solution (pH = 10.5) (see Experimental Section). Prior to the NRR experiments, the as-synthesized AgNDs were first mixed with Ar-saturated 0.1 M Na_2_SO_4_ solution (pH = 10.5), then transferred to the electrochemical cell to perform the NRR measurements. It should be noted that the used Ar and N_2_ (or ^15^N_2_) feeding gases need to be first pre-purified by a similar protocol as previous works reported to remove the possible interferences of NH_3_ and NO_x_ in Ar or N_2_ (or ^15^N_2_)^45,46^. Supplementary Fig. 4 shows the experimental setup. A Cu-Fe-Al catalyst (Supplementary Fig. 5) unit was used to remove NO_x_. A CrO_3_ column was used to eliminate any possible NO interference by converting NO to water-soluble NO_2_, which can then be removed by H_2_SO_4_ solution and distilled water absorption units. And the produced tail gas was absorbed by two-series tail gas absorbers (each absorber contains 20 mL of 1.0 mM H_2_SO_4_ solution) to prevent the produced NH_3_ with N_2_ flow into air during NRR^46^. Therefore, the solution samples obtained from cathodic compartment, anodic compartment and tail gas absorbers will be all determined to analyze the yielded NRR products. Figure 2a shows the linear sweep voltammetry (LSV) curves of Ti mesh with and without AgNDs in Ar- and N_2_- saturated 0.1 M Na_2_SO_4_ solutions (pH = 10.5) with a scan rate of 5.0 mV s^−1^. The results indicate that in the investigated potential range, ignorable change in the cathodic current is observable for Ti mesh without AgNDs in both Ar- and N_2_-saturated 0.1 M Na_2_SO_4_ solutions, suggesting poor NRR activity of Ti mesh. When the AgNDs were introduced to 0.1 M Na_2_SO_4_ solution with a concentration of 0.0015 mg mL^−1^, the cathodic currents are obviously increased in Ar-saturated 0.1 M Na_2_SO_4_ solution. Considering no oxidation-state Ag existence in AgNDs, the enhanced cathodic currents with applied negative potential in Ar-saturated solution are mainly attributed to the occurred hydrogen evolution reaction (HER)^47–49^. Comparatively, the cathodic currents in LSV curve are apparently increased with applied negative potential in N_2_-saturated solution with AgNDs incorporation, suggesting that the AgNDs are electrocatalytic activity toward the NRR. In this study, the yielded NRR products including possible NH_3_ and/or N_2_H_4_ were quantitatively analyzed by the indophenol blue method (Supplementary Fig. 6) and the Watt and Chrisp method (Supplementary Fig. 7)^50,51^. The preliminary experimental results confirm that only NH_3_ product can be detected and N_2_H_4_ is ignorable (Supplementary Fig. 7d). Furthermore, NH_3_ product can be detected in both cathodic and anodic compartments, undetectable in the tail gas absorbers. In subsequent experiments, the obtained NH_3_ yield were therefore calculated from the total yielded NH_3_ amount detected in the samples obtained from both cathodic and anodic compartments (Supplementary Fig. 8a–c). The detected NH_3_ in anodic compartment is mainly attributed to the diffusion of the produced NH_3_ in cathodic compartment through the proton exchange membrane (Nafion 117 in our case)^52^. Figure 2b shows the dependence of NH_3_ yield rate (denoted as R_NH3_) and faradaic efficiency (FE) on the applied potential in N_2_-saturated 0.1 M Na_2_SO_4_ solution (pH = 10.5) with AgNDs incorporation for 1 h NRR. The corresponding chronoamperometric profiles obtained at different potentials are shown in Supplementary Fig. 8d. As shown in Fig. 2b, the R_NH3_ is increased with the applied cathodic potential and the largest R_NH3_ can be achieved to be 600.4 ± 23.0 μg h^−1^ mg_Ag_^−1^ with a FE of 10.1 ± 0.7% at −0.25 V (vs. RHE). The highest FE of 16.7 ± 0.9% can be obtained at −0.15 V (vs. RHE). When the applied potential is over −0.25 V (vs. RHE), the R_NH3_ and FE are obviously decreased, mainly owing to the competitive HER process concurrently occurred on the AgNDs^25,53^. In addition, we also performed the experiment to quantify the produced H_2_ during NRR. At −0.25 V (vs. RHE) in N_2_-saturated 0.1 M Na_2_SO_4_ electrolyte (pH = 10.5) for 1 h NRR (Supplementary Fig. 9a), the amount of H_2_ produced was 778.2 μL (Supplementary Fig. 9d) calculated according to H_2_ standard curve (Supplementary Fig. 9b, c), while the amount of yielded NH_3_ was 0.93 μg mL^−1^. Based on the time-dependent current curve (Supplementary Fig. 9a), the faradaic efficiency (FE) of H_2_ produced was calculated to be ~88.3%, while the FE of NH_3_ yielded was ~10.2%. The total FE is ~98.5%. Considering the analytical errors involved, the nearly 100% total FE obtained from the measured H_2_ and NH_3_ further confirms the reported FE for NH_3_ production.

To evaluate the stability of AgNDs toward the NRR, we performed the NRR experiment at −0.25 V (vs. RHE) for 5 h. A slight decay of the cathodic current can be found in the time-current profile (Supplementary Fig. 10a), and R_NH3_ is found to be slightly decreased with increasing the time and the R_NH3_ after 5 h of NRR is measured to be 578.4 ± 22.1 μg h^−1^ mg_Ag_^−1^, indicating good NRR stability of the AgNDs in the non-loading electrocatalysis system. The corresponding R_NH3_ and NH_3_ yield measured every 1 h during 5 h NRR are shown in Supplementary Fig. 10b, c. The in situ time-dependence Raman measurements were also conducted to confirm the structural change of AgNDs during durability test. As shown in Supplementary Fig. 11, all Raman spectra of the AgNDs in N_2_-saturated 0.1 M Na_2_SO_4_ solution (pH = 10.5) with reaction time are almost identical to that of quartz glass substrate and no Raman peaks of oxidation-state Ag appear, such as Ag_2_O, meaning that the metallic Ag phase is still dominant in the AgNDs during durability test. This can be further confirmed by the TEM characterization results of AgNDs after 5 h NRR measurement (Supplementary Fig. 12), and the AgNDs are still uniformly dispersed in the solution and remain well metallic Ag nature. This also means that the abundant catalytic active sites exposed on AgNDs are still maintained well, resulting in high NRR activity during durability test. In addition, the XPS analysis results show that compared to the pristine AgNDs, besides of Ag and O elements, additional N element can be also detected for the AgNDs sample after 5 h NRR (Supplementary Fig. 13a). The high-resolution N 1 s XPS spectrum (Supplementary Fig. 13b) of AgNDs after 5 h NRR displays a peak at binding energy of ~400.2 eV, possibly owing to the formed N-intermediates (e.g., -NH_x_) during NRR^54–56^. In addition, the high-resolution Ag 3d and O 1 s XPS spectra (Supplementary Fig. 13c, d) of AgNDs after 5 h NRR have no obvious change compared to the pristine AgNDs, further demonstrating high NRR stability of AgNDs. To assure the reproductivity of a non-loading electrocatalysis system for high-efficiency NRR, another two batches of AgNDs aqueous solution were fabricated by the laser-ablation technique. The TEM and HRTEM images indicate that all AgNDs samples exhibit homogeneously dispersed nanodot morphology with the (111) plane of fcc-phase metallic Ag (Supplementary Fig. 14a, b), confirming high reproductivity of the fabricated AgNDs catalyst by the laser-ablation technique. To further confirm the reproductivity of AgNDs catalyst for high-efficiency NRR, we also performed the electrocatalytic NRR experiments using another two batches of AgNDs catalysts at −0.25 V (vs. RHE) for 1 h. The results show that the R_NH3_ of the two AgNDs catalysts can be achieved to be 604.9 ± 17.0 μg h^−1^ mg_Ag_^−1^ and 591.9 ± 25.0 μg h^−1^ mg_Ag_^−1^ with FE of 10.8 ± 0.7% and 9.8 ± 0.6% respectively (Supplementary Fig. 14c), close to those of the reported AgNDs catalyst in this work, giving a R_NH3_ of 600.4 ± 23.0 μg h^−1^ mg_Ag_^−1^ and a FE of 10.1 ± 0.7%. The above results demonstrate high reproducibility of this AgNDs-incorporated non-loading electrocatalysis system for high-efficiency NRR.

To eliminate the environmental interferences on the yielded NH_3_ during NRR, several control experiments were carried out in this work, including 0.1 M Na_2_SO_4_ solution (pH = 10.5) (blank solution), 0.1 M Na_2_SO_4_ solution (pH = 10.5) with AgNDs under open-circuit condition (open-circuit), and Ar-saturated 0.1 M Na_2_SO_4_ solution (pH = 10.5) with AgNDs at −0.25 V (vs. RHE) for 1 h (Ar-saturated solution). The results demonstrate that ignorable NH_3_ product is detectable for all cases (Supplementary Fig. 15), thus eliminating any noticeable environmental interference to the yielded NH_3_ from the AgNDs catalyzed NRR. In addition, we also compared the yielded NH_3_ amount using Ti mesh electrode without AgNDs incorporation in N_2_-saturated 0.1 M Na_2_SO_4_ solution (pH = 10.5) at −0.25 V (vs. RHE) for 1 h NRR. Obviously, inferior NRR activity can be achieved for the Ti mesh without AgNDs (Supplementary Fig. 15). Moreover, To eliminate the interferences of possible NO_x_ (including NO and NO_2_) toward on the yielded NH_3_ during NRR, ion chromatography (IC 6000, Wayeal Co. Ltd. China) measurement was performed to verify whether there is NO_x_ present at all key stages of the experiment. As shown in Supplementary Fig. 16, no detectable NO_2_^−^ and NO_3_^−^ can be observed in all sample solution, demonstrating that the yielded NH_3_ are resulted entirely from the electrocatalytic NRR process.

To further confirm the yielded NH_3_ from the AgNDs catalyzed NRR, we subsequently conducted the ^15^N isotopic labeling experiments^45,57^. The ^1^H nuclear magnetic resonance (NMR) spectra were obtained using ^14^N_2_ and ^15^N_2_ as the feeding gases in AgNDs incorporated 0.1 M Na_2_SO_4_ solution (pH = 10.5) at −0.25 V (vs. RHE) over 1 h NRR period. Figure 2c shows the ^1^H NMR spectra of the standards and the yielded ^14^NH_4_^+^ and ^15^NH_4_^+^ products in the NRR samples, confirming that the yielded NH_3_ is indeed exclusively resulted from the AgNDs catalyzed NRR. The quantitative analysis results, based on the standard ^14^NH_4_^+^ and ^15^NH_4_^+ 1^H NMR spectra and corresponding calibration curves (Supplementary Fig. 17)^25^ confirm that the concentration of the yielded ^14^NH_4_^+^ and ^15^NH_4_^+^ (normalized to the yielded NH_3_ concentration in cathodic compartment with 50 mL of electrolyte) is 0.86 and 0.92 μg mL^−1^ respectively, almost identical to the results (0.90 and 0.88 μg mL^−1^ for ^14^NH_4_^+^ and ^15^NH_4_^+^, respectively) obtained from the indophenol blue detection method. To further illustrate the advantages of the used non-loading electrocatalysis system for high-efficiency NRR, we also carried out several comparison experiments. The as-synthesized AgNDs were coated on the commercial carbon cloth (CC) substrate to prepare AgNDs/CC electrode, which was used as the cathode for NRR measurement at −0.25 V (vs. RHE) in N_2_-saturated 0.1 M Na_2_SO_4_ solution (pH = 10.5) for 1 h using a conventional catalyst-loading electrocatalysis system (Supplementary Fig. 1b). For meaningful comparison, Ag_2_O nanoparticles (Ag_2_ONPs) with an average particle size of ~13 nm (Supplementary Fig. 18) were fabricated by treating O_2_-saturated AgNDs solution at 50 °C for 1 h and Ag nanoparticles (AgNPs) with an average particle size of ~17 nm obtained by the laser-ablation technique (Supplementary Fig. 19), were also evaluated for the NRR under the identical experimental conditions. The results (Supplementary Fig. 20) show that after 1 h NRR, the AgNDs/CC, Ag_2_ONPs/CC and AgNPs/CC can give an NH_3_ yield rate of 80.1 ± 1.5, 58.0 ± 2.1 and 66.0 ± 1.3 μg h^−1^ mg_cat._^−1^ with a FE of 6.2 ± 0.1%, 4.9 ± 0.2% and 5.3 ± 0.2% at −0.25 V (vs. RHE), respectively. Among all investigated catalysts, the Ag_2_ONPs/CC exhibits the lowest NRR activity, mainly owing to an easy reductive property of Ag_2_O to consume large number of electrons but not for the NRR^35,37,38^. Comparatively, the AgNDs/CC gives higher NRR activity than that of AgNPs/CC, indicating that Ag nanodots with smaller sizes may provide more active sites for the NRR. The NH_3_ yield rate of AgNDs catalyst using non-loading electrocatalysis system is almost 7.5 times of that obtained from the AgNDs/CC using the conventional catalyst-loading electrocatalysis system. This is primarily ascribed to the Ag nanodots uniformly dispersed in solution capable of providing more catalytic active sites for NRR, while the limited loading amount of AgNDs on CC and easy aggregation to form large-sized Ag nanoparticles during NRR could result in significantly decreased NRR performance. The highly dispersed Ag nanodots in solution can supply abundant active sites^12,14,16^ for chemically adsorbing N_2_ molecules, and then these chemisorptive N_2_ molecules on AgNDs accept the attack of H^+^/e^−^ at Ti mesh to form NH_3_ molecules. This can be further verified by the N_2_ temperature-programmed desorption (TPD) measurement (Fig. 2d). Compared to the CC substrate, AgNDs/CC obviously displays a dramatically enhanced N_2_ desorption peak at ~170 °C, meaning superior adsorption capability of AgNDs toward N_2_^31^. In addition, we also investigated the influence of current collector using commercial carbon cloth (CC) to replace metallic Ti mesh in the non-loading electrocatalysis system. The results (Supplementary Fig. 21) show that the NH_3_ yield rate of AgNDs catalyst using commercial carbon cloth (CC) as the current collector can give an NH_3_ yield rate of 402.3 ± 15.0 μg h^−1^ mg_Ag_^−1^ with a FE of 16.7 ± 0.7% at −0.25 V (vs. RHE) after 1 h NRR measurement. The higher NH_3_ yield rate using metallic Ti mesh as the current collector could be due to its good electrical conductivity with superior dynamic NRR process. Furthermore, the concentration effect of AgNDs on the NH_3_ yield rate was also investigated in this work. As seen in Supplementary Fig. 22, the NH_3_ yield is linearly increased with increasing the AgNDs concentration, but the change in the NH_3_ yield rate normalized to per milligram of AgNDs is not obvious with increasing the AgNDs concentration, possibly owing to lower N_2_ solubility in aqueous solution and/or mass transport influence with higher AgNDs concentration in the non-loading electrocatalysis system.

### Ti plate-based two-electrode configured flow-type electrochemical reactor

**Fig. 3:**
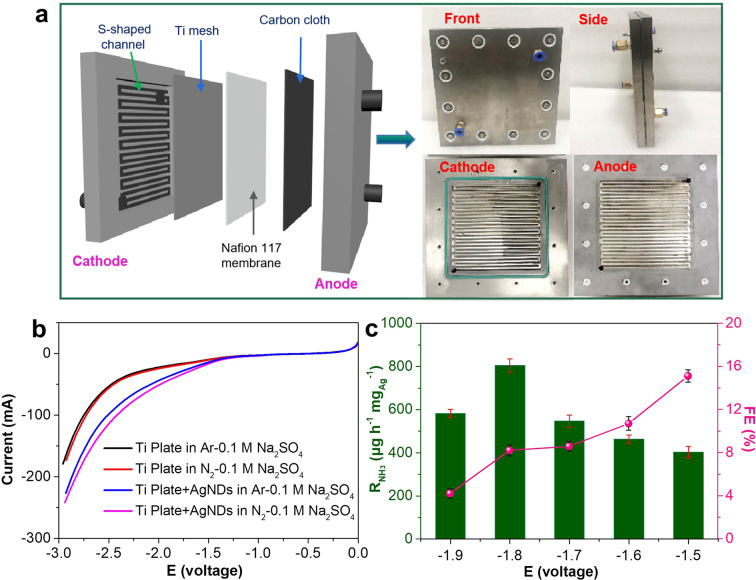
NRR evaluation of AgNDs in Ti plate-based two-electrode configured flow-type electrochemical reactor. **a** Schematic illustration of two-electrode configured flow-type electrochemical reactor and photographs of assembled reactor and individual cell components. **b** LSV curves of Ti plate-based two-electrode flow-type electrochemical reactor with and without AgNDs incorporation in Ar- and N_2_-saturated 0.1 M Na_2_SO_4_ solutions (pH = 10.5). **c** The dependence of NH_3_ yield rate and faradaic efficiency of AgNDs catalyst on the cell voltages in N_2_-saturated 0.1 M Na_2_SO_4_ electrolyte (pH = 10.5). The error bars correspond to the standard deviations of three independent measurements.

Except for high-efficient electrocatalysts, design and development of high-performance electrochemical NRR reactors are also critically important for NH_3_ production. Several reported works have verified that the flow-type electrochemical reactors are very favorable for improving the NRR performance due to efficient mass transport and high coverage of N_2_ on the catalyst^31,58,59^. In this work, a Ti plate-based two-electrode configured flow-type electrochemical reactor was therefore developed to evaluate the AgNDs’ NRR performance (Fig. 3a), which should be very suitable for high-efficient utilization of aqueous AgNDs catalyst. During NRR measurements, two Ti plates with “S-type” channel patterns were separated by the Nafion 117 membrane, moreover, a slice of Ti mesh and carbon cloth (CC) was respectively placed in cathodic compartment and anodic compartment to decrease the excessive current directly caused from the Ti plate current collector to damage Nafion 117 membrane (Fig. 3a). In the cathode side, N_2_-saturated 0.1 M Na_2_SO_4_ solution with AgNDs incorporation was circulated through the cathodic compartment and returned back to the reservoir with a flow rate of 10 mL min^−1^ under the help of peristaltic pump, while the reaction on anode was conducted at the same conditions without AgNDs incorporation. The purification of feeding gases strictly follows the reported procedure^45,46^. The LSV curves obtained in this two electrode configured flow-type electrochemical cell (Fig. 3b) indicate that the introduction of AgNDs in electrolyte can dramatically improve the NRR activity. Subsequently, we performed the chronoamperometric tests to investigate the effect of applied voltage on NH_3_ yield rate (R_NH3_) and faradaic efficiency (FE) in this flow-type electrochemical cell. The experimental results show that NH_3_ product can be detected in both cathodic and anodic compartments (undetectable in the tail gas absorbers), consistent with the results obtained in three-electrode system (Supplementary Fig. 23a, b). Figure 3c shows the dependence of R_NH3_ and FE on the applied cell voltage in N_2_-saturated 0.1 M Na_2_SO_4_ solution (pH = 10.5) with AgNDs incorporation for 1 h NRR. The corresponding chronoamperometric curves are shown in Supplementary Fig. 23c. It can be seen that the highest FE of 15.1 ± 0.6% can be obtained at an voltage of −1.5 V, then decreased at more negative voltage. The largest R_NH3_ is achieved to be 804.5 ± 30.6 μg h^−1^ mg_Ag_^−1^ with a FE of 8.2 ± 0.5% at −1.8 V. Obviously, the obtained NH_3_ yield rate in this flow-type electrochemical cell is higher than that from the three-electrode configured system, demonstrating the superiority of aqueous AgNDs-incorporated flow-type reactor.

## Discussion

The above experimental results have illustrated that it is very feasible for high-efficiency electrocatalytic NRR to NH_3_ utilizing AgNDs introduced three-electrode and two-electrode electrochemical systems. Even so, the faradaic efficiencies of these reaction systems are not very high, and the NRR active mechanism on AgNDs catalyst needs to be clarified in this work.

### Improvement of NRR performance

**Fig. 4:**
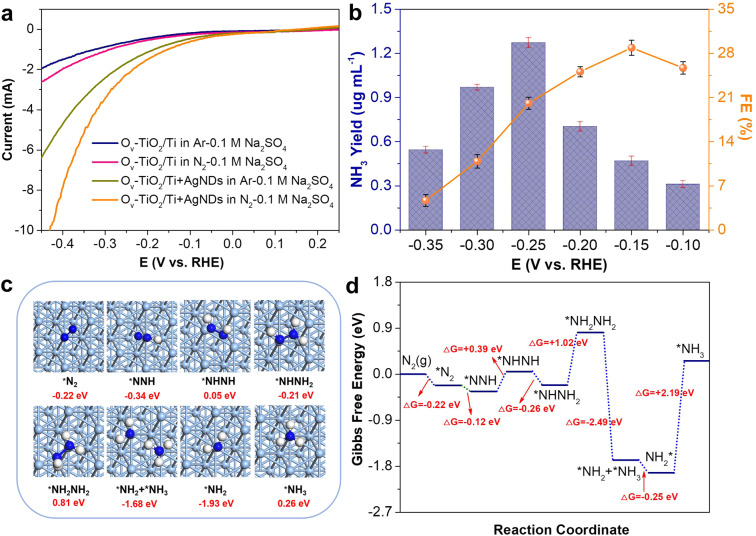
Improvement of NRR performance and NRR active mechanism of AgNDs. **a** LSV curves of O_v_-TiO_2_/Ti with and without AgNDs incorporation in Ar- and N_2_-saturated 0.1 M Na_2_SO_4_ solutions (pH = 10.5). **b** The dependence of NH_3_ yield and faradaic efficiency of AgNDs catalyst on the applied potential using O_v_-TiO_2_/Ti current collector. The error bars correspond to the standard deviations of three independent measurements. **c** The top view of the optimized structures of Ag (111) plane along the lowest energy NRR reaction pathway for N_2_ conversion into NH_3_ (Ag: cyan, N: blue, and H: white). **d** Free-energy diagram of the N_2_ reduction reaction at the optimized Ag (111) plane.

Based on Fig. 2a, the AgNDs introduction into Ar- or N_2_-saturated electrolyte results in significantly improved cathodic current in the three-electrode configured electrocatalysis system using metallic Ti mesh as the current collector. The majority of the enhanced cathodic current is primarily originated from the superior hydrogen evolution reaction (HER) of AgNDs^16,60^ on metallic Ti mesh with high electrical conductivity, which results in a decreased faradaic efficiency (FE) of electrocatalytic NRR. To improve the FE of NRR with high NH_3_ yield, we speculated that the modification of a metal oxide layer on metallic Ti mesh could be an effective solution to decrease the cathodic current and thus the competitive HER process. The modified metal oxide layer can not only effectively reduce the cathodic current of metallic Ti mesh during AgNDs-incorporated NRR, but also possibly serve as the electrocatalyst providing catalytic active sites for enhanced NRR performance. For this, we fabricated oxygen vacancies-rich TiO_2_ modified metallic Ti mesh (denoted as O_v_-TiO_2_/Ti, see Experimental Section) as the current collector for NRR evaluation. The X-ray diffraction (XRD) patterns (Supplementary Fig. 24) of O_v_-TiO_2_/Ti show that the modified TiO_2_ on metallic Ti mesh is anatase phase (JCPDS No. 21 − 1272). The scanning electron microscopy (SEM) images of pristine metallic Ti mesh and O_v_-TiO_2_/Ti samples indicate that no obvious nanostructures can be observed for pristine Ti mesh (Supplementary Fig. 25a–c), while rougher surface structure consisted of nanosheets is clearly observable (Supplementary Fig. 25d–f) for O_v_-TiO_2_/Ti. The TEM characterization was further used to precisely analyze the fabricated TiO_2_ nanosheet structure. As shown in Supplementary Fig. 26, nanosheet structure is obviously dominant and the lattice spacing of 0.352 nm of an individual nanosheet in HRTEM image is corresponding to the (101) plane of anatase TiO_2_. Interestingly, it was found that there are some disordered layers on the edge of TiO_2_ nanosheet, possibly owing to the introduction of rich oxygen vacancies^61^. This can be further verified by the electron paramagnetic resonance (EPR) spectra (Supplementary Fig. 27), in where a stronger signal peak at around g = 2.007 is primarily originated from the generated rich oxygen vacancies in TiO_2_ nanosheets of O_v_-TiO_2_/Ti. Recently, our and other groups have demonstrated that oxygen vacancies in metal oxides catalysts can be used as the catalytic active sites for N_2_ adsorption, activation and hydrogenation^55,61–63^. In this work, O_v_-TiO_2_/Ti can concurrently serve as the current collector and NRR electrocatalyst bifunctionality. As expected, in comparison with metallic Ti current collector (Fig. 2a), the electrocatalysis system using O_v_-TiO_2_/Ti exhibits significantly decreased cathodic current in Ar- and N_2_-saturated Na_2_SO_4_ electrolyte with or without AgNDs incorporation (Fig. 4a). Moreover, the O_v_-TiO_2_/Ti shows good electrocatalytic NRR activity (Fig. 4a) in N_2_-saturated electrolyte. Figure 4b displays the dependence of NH_3_ yield (normalized to the yielded NH_3_ concentration in cathodic compartment with 50 mL of electrolyte) and faradaic efficiency (FE) on the applied potential in the non-loading electrocatalysis system using O_v_-TiO_2_/Ti current collector. The corresponding UV-Vis absorption spectra and chronoamperometric curves are shown in Supplementary Fig. 28. As illustrated, the highest FE can be obtained to be 28.9 ± 1.2% at −0.15 V (vs. RHE) with 1 h NRR, while the largest NH_3_ yield can be achieved to be 1.27 ± 0.03 μg mL^−1^ with a FE of 20.1 ± 0.9% at −0.25 V (vs. RHE) for 1 h NRR, which includes two part contributions from O_v_-TiO_2_/Ti and AgNDs catalyzed NRR processes. For O_v_-TiO_2_/Ti catalyzed NRR without AgNDs, the NH_3_ yield can be obtained to be 0.37 ± 0.01 μg mL^−1^ with a FE of 10.1 ± 0.8% at −0.25 V (vs. RHE) for 1 h NRR (Supplementary Fig. 29). Apparently, the introduction of AgNDs results in significantly improved NRR performance using O_v_-TiO_2_/Ti current collector with high stability (Supplementary Fig. 30), primarily owing to superior NRR activity of AgNDs combined with the contribution from O_v_-TiO_2_/Ti catalyzed NRR. In addition, the NH_3_ yield and FE in the non-loading electrocatalysis system using O_v_-TiO_2_/Ti current collector are almost 1.4 and 2 times of those achieved from the electrocatalysis system using metallic Ti mesh current collector (NH_3_ yield of 0.90 ± 0.03 μg mL^−1^ and FE of 10.1 ± 0.7% at −0.25 V vs. RHE), demonstrating that the strategy of metallic Ti mesh modification is feasible for further enhancing the electrocatalytic NRR performance.

### NRR active mechanism

A recent work has demonstrated that the catalytic activity of Ag nanoparticles is dramatically enhanced with decreasing the nanoparticle size to ~5 nm^12^. In addition, the theoretical and experimental results have revealed that the (111) planes exposed Ag nanostructures possess high electrocatalytic activities^14,16,27,28^. In this work, the fabricated aqueous Ag nanodots with small sizes exhibit highly exposed (111) crystalline planes (Fig. 1b, Supplementary Fig. 12), we therefore constructed the optimized structure model of Ag(111) plane (Supplementary Fig. 31) to investigate the NRR active mechanism of AgNDs by the density functional theory (DFT) calculations. Firstly, we optimized the adsorption configurations of N_2_ on the Ag(111) plane. As shown in Supplementary Fig. 32, an end-on adsorption configuration with a certain inclination was found to be more thermodynamically favorable for N_2_ adsorption and activation. The DFT calculations results indicate that N-N bond can be elongated to 1.114 Å for the adsorbed N_2_ on Ag(111) plane from 1.090 Å for an isolated N_2_ molecule, suggesting the possibility of the first hydrogenation step in the next step. Figure 4c presents the top view of the optimized structures of Ag(111) surface along the lowest energy NRR reaction pathway for N_2_ conversion to NH_3_. After the first hydrogenation of N_2_, the N-N bond is elongated from 1.114 Å of *N_2_ to 1.180 Å of *N_2_H. The N-N bond length can be further elongated to 1.260 Å of *NHNH, 1.358 Å of *NHNH_2_ and 1.431 Å of *NH_2_NH_2_ by an alternative hydrogenation process. At last, the N ≡ N triple bond breaks to form the first NH_3_ molecule. Based on our theoretical calculations, the 1^st^, 3^rd^, 5^th^ and 6^th^ hydrogenation steps are exothermic processes and the energies of 2^nd^, 4^th^ and 7^th^ (second NH_3_ generation) hydrogenation steps are up-hill (Fig. 4d). Moreover, the formation of second NH_3_ molecule (7^th^ hydrogenation step) can be identified as the rate determining step with a needed energy (△G) of 2.19 eV. The above theoretical calculations results indicate that the Ag(111) surface is capable of adsorbing and activating N_2_ molecules, thus affording excellent NRR performance.

In summary, we demonstrated the feasibility of using Ag nanodots catalyst dispersed in aqueous solution in non-loading electrocatalysis system for high-efficiency electrocatalytic N_2_ reduction reaction (NRR) to NH_3_. Such electrochemical system can take full advantage of the catalytic active sites provided by Ag nanodots with highly exposed (111) planes for N_2_ adsorption and activation, and the NRR performance can be further enhanced by simple modification of the metallic Ti mesh current collector. Using the fabricated aqueous Ag nanodots catalyst, a two-electrode configured flow-type electrochemical reactor has been developed and evaluated for the NRR, demonstrating great potential for NH_3_ production. Our work provides a significant guidance on designing high-efficiency electrocatalysts and electrocatalysis systems for ambient electrosynthesis of NH_3_.

## Methods

### Fabrication of Ag nanodots (AgNDs) solution

Aqueous AgNDs solution was synthesized by a facile one-step laser-ablation technique. In a typical synthesis, a polished silver metal plate (99.99% purity) was fixed on a bracket in a vessel containing 17 mL of deionized water under continuously stirring (10 rpm) by an amotorized tunable stage (WNSC 400). The deionized water level above the silver target surface was ~10 mm. The silver plate in deionized water was subsequently irradiated for 30 min by a focused laser (532 nm Nd:YAG pulsed laser) with 8 ns pulse duration, 100 mJ power energy per pulse and spot size of 1.0 mm in diameter in Ar atmosphere. Finally, the AgNDs solution was obtained with a color of bright yellow. Ag_2_O nanoparticles (Ag_2_ONPs) solution was fabricated from the O_2_-saturated AgNDs solution at 50 °C for 1 h.

### Fabrication of Ag nanoparticles (AgNPs) solution

AgNPs solution was synthesized employing the similar progress with AgNDs solution fabrication using the laser-ablation technique. In a typical synthesis, the polished silver metal plate was ablated in 15 mL of deionized water with a focused laser at 1064 nm, 80 mJ power energy per pulse and spot size of 1.5 mm in diameter under Ar atmosphere to obtain AgNPs solution.

### Fabrication of oxygen vacancies-rich TiO_2_ nanosheets modified metallic Ti mesh

In a typical synthesis, metallic titanium (Ti) mesh (4.0 × 4.0 cm^2^) was placed in a Teflon-lined stainless steel autoclave (50 mL, Anhui Kemi Machinery Technology Co., Ltd) with 30 mL of 5.0 M NaOH solution for hydrothermal reaction at 180 °C with 14 h. After the autoclave was cooled down to room temperature, the obtained samples were collected and washed with deionized water and ethanol for several times, and dried in an oven at 60 °C for 20 min. Then the samples were immersed into 0.5 M HCl solution for 2 h to exchange Na^+^ with H^+^. Finally, the oxygen vacancies-rich TiO_2_ nanosheets modified metallic Ti mesh (O_v_-TiO_2_/Ti) was achieved by thermal treatment of the hydrothermally obtained sample at 700 °C in H_2_/Ar atmosphere for 1 h with a heating rate of 5 °C min^−1^.

### Characterization

The morphologies and precise structures of the samples were determined by the field emission scanning electron microscopy (FESEM, Quanta 200FEG) and transmission electron microscopy (TEM, JEOL JEM-2010 and Tecnai TF20 TMP). The X-ray photoelectron spectroscopy (XPS) analysis of the samples was performed on an ESCA LAB250 X-ray photoelectron spectrometer (Thermo, America) equipped with A1 Kα as the X-ray source. The Raman spectra of the samples were recorded on a RXN1-785 Raman spectrometer (Analytik Jena AG, excited wavelength of 785 nm). For in situ Raman tests, the experiments were performed on RXN1-785 Raman spectrometer connected with CHI660D electrochemical workstation. A quartz-made electrochemical cell containing AgNDs nanodots electrocatalyst dispersed in 0.1 M Na_2_SO_4_ solution (pH = 10.5) was placed in the front of Raman fiber optic probe. Potentiostatic tests were conducted following the same way in long time durability test at −0.25 V (vs. RHE) for 5 h NRR and Raman spectra with temporal resolution of 30 s was adopted to achieve the in situ monitoring. The amount of Ag nanodots in solution was analyzed by the inductively coupled plasma optical emission spectroscopy (ICP-OES 6300, Thermo Fisher Scientific). The N_2_ temperature-programmed desorption (N_2_-TPD) experiments were conducted on a Quantachrome ChemBET Pulsar TPR/TPD. The Zeta potential of the Ag nanodots solution was measured using JS94H2 micro-electrophoresis instrument (Shanghai Zhongchen, China). The room temperature electron paramagnetic resonance (EPR) spectra were recorded on the Steady High Magnetic Field Facilities, High Magnetic Field Laboratory, CAS.

### Electrochemical measurements

All electrochemical measurements were performed at room temperature on CHI660D (CH Instruments, Inc., Shanghai, China) electrochemical workstation using a three-electrode configured two-compartment electrochemical cell (ZY-CD02A, Chintek Instrument & Equipment Co., Ltd. China). In the non-loading electrocatalysis system, metallic Ti mesh or O_v_-TiO_2_/Ti (4.0 × 4.0 cm^2^) was used as the current collector, and commercial carbon cloth and Ag/AgCl (saturated KCl electrolyte) were used as the counter electrode and reference electrode, respectively. Na_2_SO_4_ powder was thermally treated under 5% Ar stream before preparation of 0.1 M Na_2_SO_4_ solution. The synthesized AgNDs solution (3.0 mL, 0.075 mg, pH = 9.5) as the electrocatalyst was dispersed into Ar-saturated 0.1 M Na_2_SO_4_ solution (47 mL) in the cathodic compartment for NRR measurements. The pH value of 0.1 M Na_2_SO_4_ solution containing the synthesized AgNDs solution with different concentrations were all adjusted to 10.5. For comparison, the conventional catalyst-loading electrocatalysis experiments were also performed using AgNDs (or AgNPs, Ag_2_ONPs) coated on commercial carbon cloth (CC) as the working electrode, commercial CC as the counter electrode, and Ag/AgCl (saturated KCl electrolyte) as the reference electrode. For the preparation of working electrode, 3.0 mL of AgNDs (or AgNPs, Ag_2_ONPs) solution containing 5.0 wt.% Nafion solution was first ultrasonically treated for 30 min to form a homogeneous catalyst ink, and then the catalyst ink was casted on a clean carbon cloth (1.0 × 1.0 cm^2^) with a catalyst loading amount of ~0.075 mg cm^−2^ and subsequently dried at 60 °C for 2 h in vacuum. Both of the cathodic chamber and anodic chamber contained 50 mL of 0.1 M Na_2_SO_4_ solution (pH = 10.5). In this work, all measured electrochemical potentials were calibrated to be vs. reversible hydrogen electrode (RHE) by the following equation:

$$E({\mathrm{V}}\,{\mathrm{vs}}{\mathrm{.RHE}}) = E({\mathrm{V}}\,{\mathrm{vs}}.{\mathrm{Ag}}/{\mathrm{AgCl}}) + 0.059 \times {\mathrm{pH}} + 0.197$$

where *E* (V vs. RHE) is the converted potential (V) vs. RHE, *E* (V vs. Ag/AgCl) is the experimentally measured potential against Ag/AgCl reference electrode, 0.197 is the value of standard potential for the Ag/AgCl reference electrode (saturated KCl electrolyte) at 25 °C.

For the two-electrode system NRR evaluation, a metallic Ti plate-based flow-type electrochemical reactor was designed and developed, and Nafion 117 membrane was sandwiched by a piece of carbon cloth (10 × 10 cm^2^) and metallic Ti mesh (10 × 10 cm^2^), and then all clamped together using two metallic Ti plates with S-shaped channel served as the current collectors. For a typical electrochemical test in this flow-type cell, 50 mL of 0.1 M Na_2_SO_4_ solution (pH = 10.5) with and without 0.003 mg mL^−1^ AgNDs was respectively flowed through the cathodic cell and anodic cell, and then returned back to the reservoir using multichannel peristaltic pump at a flow rate of 10 mL min^−1^. The electrolyte solutions after NRR tests were extracted in the reservoir for further identification and quantification.

The polarization curves during electrochemical measurements were recorded with a scan rate of 5.0 mV s^−1^ at room temperature and all polarization curves were obtained at the steady-state ones after several cycles. For the non-loading electrocatalysis measurements and two-electrode flow-type cell system, the potentiostatic tests were conducted in N_2_-saturated 0.1 M Na_2_SO_4_ solution (pH = 10.5) containing AgNDs with continuously bubbling N_2_ at a flow rate of ~15 mL min^−1^ under adequately stirring at different potentials for 1 h. All NRR experiments were carried out at room temperature and atmospheric pressure. Prior to each measurement, N_2_ or Ar feeding gas was first pre-purged by the Cu-Fe-Al catalyst, CrO_3_ column, 1.0 mM H_2_SO_4_ solution (20 mL) and distilled water (20 mL) to eliminate the potential NO_x_ and NH_3_ contaminants based on the previously reported protocols^45,46^.

### Determination of ammonia

The produced ammonia during NRR was spectrophotometrically detected by the indophenol blue method on a UV-Vis 2700 spectrophotometer (Shimadzu, Japan). In detail, 5.0 mL of sample was diluted with 5.0 mL of deionized water. Subsequently, 500 μL of 0.55 M NaOH coloring solution (5.0 wt.% salicylic acid and 5.0 wt.% sodium citrate), 100 μL of catalyst solution (0.1 g Na_2_[Fe(CN)_5_NO]·2H_2_O diluted to 10 mL with deionized water), 100 μL of oxidizing solution (sodium hypochlorite (ρCl = 4~4.9) and 0.75 M sodium hydroxide) were added respectively to the measured sample solution. After standing at room temperature for 1 h, the UV-Vis absorption spectrum was measured at a wavelength of 697.5 nm. The concentration-absorbance curves were calibrated using standard NH_4_Cl solutions with a series of concentrations in 0.1 M Na_2_SO_4_ solution (pH = 10.5) with and without AgNDs incorporation and the obtained calibration curve (*y* = 0.013 + 0.811*x*, R^2^ = 0.999) was used to calculate the produced ammonia concentration.

### Determination of hydrazine

The produced hydrazine concentration was determined by the method of Watt and Chrisp. In detail, a mixture of para(dimethylamino) benzaldehyde (5.99 g), HCl (concentrated, 30 mL) and ethanol (300 mL) were used as the color reagent. 100 μL of sample was acidized with 10 mL of 1.0 M HCl solution, and then 5.0 mL of color reagent added to the above sample solution with rapid stirring for several times. After standing at room temperature for 20 min, the UV-Vis absorption spectrum was obtained at a wavelength of 455 nm. The concentration-absorbance curves were calibrated using standard N_2_H_4_·H_2_O solutions with a series of concentrations in 0.1 M Na_2_SO_4_ solution (pH = 10.5) with and without AgNDs incorporation, and the obtained calibration curve (*y* = 0.826*x* + 0.007, *R*^2^ = 0.999) was used to calculate the ammonia concentration.

### Calculations of NH_3_ yield rate (R_NH3_) and faradaic efficiency (FE)

The calculation of NH_3_ yield rate (*R*_NH3_) is as following equation:

$$R_{{\mathrm{NH}}_{\mathrm{3}}}\left( {{\upmu g}}\,{\mathrm{h}}^{ - {\mathrm{1}}\,{\mathrm{mg}}_{{\mathrm{cat}}{\mathrm{.}}}^{ - {\mathrm{1}}}} \right) = \frac{{\chi \left( {{\upmu g}}\,{\mathrm{mL}}^{ - {\mathrm{1}}} \right) \times V\left( {{\mathrm{mL}}} \right)}}{{t\left( {\mathrm{h}} \right) \times m\,\left( {{\mathrm{mg}}^{ - {\mathrm{1}}}} \right)}}$$

The calculation of faradaic efficiency (FE) is as following equation:

$$FE({\mathrm{\% }}) = \frac{{3 \times \chi \left( {{\upmu g}}\,{\mathrm{mL}}^{ - {\mathrm{1}}} \right) \times V\left( {{\mathrm{mL}}} \right) \times 10^{ - 6} \times F}}{{17 \times Q}} \times 100{\mathrm{\% }}$$

where *χ* (μg mL^−1^) is the produced ammonia concentration; *V* (mL) is the electrolyte solution volume; *m* is the catalyst weight; *t* (s) is the reaction time; *F* is the faradaic constant (96485.34); *Q* is the total charge passed through the electrode during NRR.

### Isotope labeling experiments

The ^14^N and ^15^N isotopic labeling experiments were conducted using ^14^N_2_ and ^15^N_2_ as the feeding gases (99% enrichment of ^15^N in ^15^N_2_ feeding gas, Supplied by Hefei Ninte Gas Management Co., LTD). Prior to use, ^14^N_2_ and ^15^N_2_ feeding gases were pre-purged by the Cu-Fe-Al catalyst, CrO_3_ column, 1.0 mM H_2_SO_4_ solution (20 mL) and distilled water (20 mL) to eliminate the potential NO_x_ and NH_3_ contaminants based on the reported protocols^45,46^. After the electrochemical reaction at −0.25 V (*vs*. RHE) for 1 h, the reaction solution of both cathodic and anodic chambers (100 mL) was concentrated to 2.0 mL at 80 °C. Then, 1.0 mL of above solution mixed with 0.2 mL of D_2_O was used for ^1^H NMR spectroscopy measurement (Bruker AVANCE AV III 400). The ^1^H NMR analysis and calibration curve construction were carried out in accordance with the reported method.

### Computational methods

To investigate the N_2_ reduction reaction (NRR) process on the Ag (111) surface, the density functional theory (DFT) calculations were carried out by Viena ab initio software package (VASP)^64,65^. The Projector Augmented Wave (PAW) potentials were used for the treatment of core electrons^66^. The generalized gradient approximation (GGA) with the Perdew-Burke-Ernzerh (PBE) function was used for description of the electron exchange correlation interactions^67^. Van der Waals interactions were described via DFT-D3 correlation^68^. A 2 × 2 × 2 supercell slab model was built with the lattice parameters of *a* = 11.64, *b* = 11.64 Å and a vacuum of 15 Å was added in the *z* direction. The convergence criterion of geometry relaxation was 0.01 eV Å^−1^in forceon each atom. The energy cutoff for plane wave-basis was set to 500 eV. The *K* points were sampled with 3 × 3 × 1 by Monkhorst-Pack method. The free energies of the NRR steps were calculated by *ΔG* = *ΔE*_ads_ + *ΔΕ*_ZPE_–*TΔS* + *ΔG*(*U*) + *ΔG*(*pH*), where *ΔE*_ads_ is the adsorption energy, *ΔΕ*_ZPE_ is the zero point energy and *S* is the entropy at 298 K. Considering the applied potential of the NRR reaction, the free energy of each step was calculated by adding the value of *ΔG*(*U*) = −*neU*, where *U* is the applied bias, *n* is the number of electrons involved in the reaction. In our calculations, we used *U* = −0.25 V (vs. RHE). *ΔG*(*pH*) = −*k*_*B*_*T*ln10 × *pH*, where *k*_*B*_ is the Boltzmann constant, and *pH* = 10.5 of the used electrolyte. In this study, the entropies of molecules in the gas phase are obtained from the literature^69^.

## Supplementary information

Supplementary Information

## Data Availability

The authors declare that all the data supporting the findings of this study are available within the article (and Supplementary Information Files), or available from the corresponding author on reasonable request.

## References

[1] Chen JG (2018). Beyond fossil fuel-driven nitrogen transformations. Science.

[2] Chu S, Majumdar A (2012). Opportunities and challenges for a sustainable energy future. Nature.

[3] Falcone M, Chatelain L, Scopelliti R, Živković I, Mazzanti M (2017). Nitrogen reduction and functionalization by a multimetallic uranium nitride complex. Nature.

[4] Légaré M-A (2018). Nitrogen fixation and reduction at boron. Science.

[5] Wang L (2018). Greening ammonia toward the solar ammonia refinery. Joule.

[6] Guo C, Ran J, Vasileff A, Qiao SZ (2018). Rational design of electrocatalysts and photo(electro)catalysts for nitrogen reduction to ammonia (NH_3_) under ambient conditions. Energy Environ. Sci..

[7] Shipman MA, Symes MD (2017). Recent progress towards the electrosynthesis of ammonia from sustainable resources. Catal. Today.

[8] Deng J, Iñiguez JA, Liu C (2018). Electrocatalytic nitrogen reduction at low temperature. Joule.

[9] Suryanto BHR (2019). Challenges and prospects in the catalysis of electroreduction of nitrogen to ammonia. Nat. Catal..

[10] Wan Y, Xu J, Lv R (2019). Heterogeneous electrocatalysts design for nitrogen reduction reaction under ambient conditions. Mater. Today.

[11] Cui X, Tang C, Zhang Q (2018). A review of electrocatalytic reduction of dinitrogen to ammonia under ambient conditions. Adv. Energy Mater..

[12] Salehi-Khojin A (2013). Nanoparticle silver catalysts that show enhanced activity for carbon dioxide electrolysis. J. Phys. Chem. C..

[13] Yang XF (2013). Single-atom catalysts: a new frontier in heterogeneous catalysis. Acc. Chem. Res..

[14] Back S, Yeom MS, Jung Y (2015). Active sites of Au and Ag nanoparticle catalysts for CO_2_ electroreduction to CO. ACS Catal..

[15] Zhang H, Liu G, Shi L, Ye J (2018). Single-atom catalysts: emerging multifunctional materials in heterogeneous catalysis. Adv. Energy Mater..

[16] Kang WJ (2019). Ultrafine Ag nanoparticles as active catalyst for electrocatalytic hydrogen production. ChemCatChem.

[17] Yan Y (2019). Recent advances on graphene quantum dots: from chemistry and physics to applications. Adv. Mater..

[18] Weiss EA (2017). Designing the surfaces of semiconductor quantum dots for colloidal photocatalysis. ACS Energy Lett..

[19] Wu HL (2019). Semiconductor quantum dots: an emerging candidate for CO_2_ photoreduction. Adv. Mater..

[20] Chu K (2019). Efficient electrocatalytic N_2_ reduction on CoO quantum dots. J. Mater. Chem. A.

[21] Wang F (2019). CuO/Graphene nanocomposite for nitrogen reduction reaction. ChemCatChem.

[22] Chu K (2019). NiO nanodots on graphene for efficient electrochemical N_2_ reduction to NH_3_. ACS Appl. Energy Mater..

[23] Liu YP (2019). ZnO quantum dots coupled with graphene toward electrocatalytic N_2_ reduction: experimental and DFT investigations. Chem. Eur. J..

[24] Chu K (2019). Electronically coupled SnO_2_ quantum dots and graphene for efficient nitrogen reduction reaction. ACS Appl. Mater. Interfaces.

[25] Cheng H (2018). Molybdenum carbide nanodots enable efficient electrocatalytic nitrogen fixation under ambient conditions. Adv. Mater..

[26] Huang H (2018). Ag nanosheets for efficient electrocatalytic N_2_ fixation to NH_3_ under ambient conditions. Chem. Commun..

[27] Ji L (2018). Nanostructured bromide-derived Ag film: an efficient electrocatalyst for N_2_ reduction to NH_3_ under ambient conditions. Inorg. Chem..

[28] Gao WY (2019). Morphology-dependent electrocatalytic nitrogen reduction on Ag triangular nanoplates. Chem. Commun..

[29] Lee HK (2018). Favoring the unfavored: selective electrochemical nitrogen fixation using a reticular chemistry approach. Sci. Adv..

[30] Yu H (2019). Bimetallic Ag_3_Cu porous networks for ambient electrolysis of nitrogen to ammonia. J. Mater. Chem. A.

[31] Chen Y (2020). Highly productive electrosynthesis of ammonia by admolecule-targeting single Ag sites. ACS Nano.

[32] Zhang D (2017). Laser synthesis and processing of colloids: fundamentals and applications. Chem. Rev..

[33] Zhao Z (2017). Vertically aligned MoS_2_/Mo_2_C hybrid nanosheets grown on carbon paper for efficient electrocatalytic hydrogen evolution. ACS Catal..

[34] Sun F (2018). NiFe-based metal-organic framework nanosheets directly supported on nickel foam acting as robust electrodes for electrochemical oxygen evolution reaction. Adv. Energy Mater..

[35] Nazemi M, El-Sayed MA (2019). The role of oxidation of silver in bimetallic gold–silver nanocages on electrocatalytic activity of nitrogen reduction reaction. J. Phys. Chem. C..

[36] Wang M (2019). Over 56.55% faradaic efficiency of ambient ammonia synthesis enabled by positively shifting the reaction potential. Nat. Commun..

[37] Nazemi M, El-Sayed MA (2019). Plasmon-enhanced photo(electro)chemical nitrogen fixation under ambient conditions using visible light responsive hybrid hollow Au-Ag_2_O nanocages. Nano Energy.

[38] Mistry H (2017). Enhanced carbon dioxide electroreduction to carbon monoxide over defect-rich plasma-activated silver catalysts. Angew. Chem. Int. Ed..

[39] Wachs IE, Roberts CA (2010). Monitoring surface metal oxide catalytic active sites with raman spectroscopy. Chem. Soc. Rev..

[40] Ling XY (2013). Alumina-coated Ag nanocrystal monolayers as surfaceenhanced raman spectroscopy platforms for the direct spectroscopic detection of water splitting reaction intermediates. Nano Res..

[41] Martina I (2012). Micro-raman characterisation of silver corrosion products: instrumental set up and reference database. e-Preserv. Sci..

[42] Xiao J, Liu P, Wang CX, Yang GW (2017). External field-assisted laser ablation in liquid: an efficient strategy for nanocrystal synthesis and nanostructure assembly. Prog. Mater. Sci..

[43] Liu Y (2015). Coupling Mo_2_C with nitrogen-rich nanocarbon leads to efficient hydrogen-evolution electrocatalytic sites. Angew. Chem., Int. Ed..

[44] Huang LB (2018). Self-limited on-site conversion of MoO_3_ nanodots into vertically aligned ultrasmall monolayer MoS_2_ for efficient hydrogen evolution. Adv. Energy Mater..

[45] Andersen SZ (2019). A rigorous electrochemical ammonia synthesis protocol with quantitative isotope measurements. Nature.

[46] Zhang S (2020). Electrocatalytically active Fe-(O-C_2_)_4_ single-Atom sites for efficient reduction of nitrogen to ammonia. Angew. Chem. Int. Ed..

[47] Wang J (2018). Ambient ammonia synthesis via palladium-catalyzed electrohydrogenation of dinitrogen at low overpotential. Nat. Commun..

[48] Strmcnik D (2016). Design principles for hydrogen evolution reaction catalyst materials. Nano Energy.

[49] Zheng Y, Jiao Y, Jaroniec M, Qiao SZ (2015). Advancing the electrochemistry of the hydrogen-evolution reaction through combining experiment and theory. Angew. Chem. Int. Ed..

[50] Searle PL (1984). The berthelot or indophenol reaction and its use in the analytical chemistry of nitrogen. Analyst.

[51] Watt GW, Chrisp JD (1952). Spectrophotometric method for determination of hydrazine. Anal. Chem..

[52] Li L (2019). Two-dimensional mosaic bismuth nanosheets for highly selective ambient electrocatalytic nitrogen reduction. ACS Catal..

[53] Bao D (2017). Electrochemical reduction of N_2_ under ambient conditions for artificial N_2_ fixation and renewable energy storage using N_2_/NH_3_ cycle. Adv. Mater..

[54] Zhao C (2019). Ambient electrosynthesis of ammonia on a biomass-derived nitrogen-doped porous carbon electrocatalyst: contribution of pyridinic nitrogen. ACS Energy Lett..

[55] Zhang S (2019). Cu doping in CeO_2_ to form multiple oxygen vacancies for dramatically enhanced ambient N_2_ reduction performance. Chem. Commun..

[56] Li W (2018). Nitrogen-free commercial carbon cloth with rich defects for electrocatalytic ammonia synthesis under ambient conditions. Chem. Commun..

[57] Greenlee LF, Renner JN, Foster SL (2018). The use of controls for consistent and accurate measurements of electrocatalytic ammonia synthesis from dinitrogen. ACS Catal..

[58] Chen S (2017). Room-temperature electrocatalytic synthesis of NH_3_ from H_2_O and N_2_ in a gas–liquid–solid three-phase reactor. ACS Sustain. Chem. Eng..

[59] Chen S (2017). Electrocatalytic synthesis of ammonia at room temperature and atmospheric pressure from water and nitrogen on a carbon-nanotube-based electrocatalyst. Angew. Chem. Int. Ed..

[60] Li Z (2019). A silver catalyst activated by stacking faults for the hydrogen evolution reaction. Nat. Catal..

[61] Han Z (2019). Activated TiO_2_ with tuned vacancy for efficient electrochemical nitrogen reduction. Appl. Catal. B: Environ..

[62] Sun Z (2019). Oxygen vacancy enables electrochemical N_2_ fixation over WO_3_ with tailored structure. Nano Energy.

[63] Zhang G, Ji Q, Zhang K (2019). Triggering surface oxygen vacancies on atomic layered molybdenum dioxide for a low energy consumption path toward nitrogen fixation. Nano Energy.

[64] Kresse G (1996). Efficiency of ab-initio total energy calculations for metals and semiconductors using a plane-wave basis set. Comp. Mater. Sci..

[65] Kresse G (1996). Efficient iterative schemes for ab initio total-energy calculations using a plane-wave basis set. Phys. Rev. B.

[66] Blöchl PE (1994). Projector augmented-wave method. Phys. Rev. B.

[67] Perdew JP (1992). Atoms, molecules, solids, and surfaces: applications of the generalized gradient approximation for exchange and correlation. Phys. Rev. B.

[68] Grimme S (2010). A consistent and accurate ab initio parametrization of density functional dispersion correction (DFT-D) for the 94 elements H-Pu. J. Chem. Phys..

[69] Wang P (2016). Breaking scaling relations to achieve low-temperature ammonia synthesis through LiH-mediated nitrogen transfer and hydrogenation. Nat. Chem..

