# Supplementary material for: Efficient electrocatalytic nitrogen reduction to ammonia with aqueous silver nanodots
Source: Commun Chem. 2021 Jan 29;4:10. doi: 10.1038/s42004-021-00449-7 (PMC9814735; doi:10.1038/s42004-021-00449-7)
Supplement: Supplementary file 1 — Supplementary Information [file 42004_2021_449_MOESM1_ESM.pdf]

Supporting Information for

Efficient electrocatalytic nitrogen reduction to ammonia with aqueous silver nanodots

Wenyi Li[1,2], Ke Li[1], Yixing Ye[1], Shengbo Zhang[1], Yanyan Liu[1,2], Guozhong Wang[1], Changhao Liang*[,1] Haimin Zhang*[,1] & Huijun Zhao[3]

[1] Key Laboratory of Materials Physics, Centre for Environmental and Energy Nanomaterials, Anhui Key Laboratory of Nanomaterials and Nanotechnology, CAS Center for Excellence in Nanoscience, Institute of Solid State Physics, HFIPS, Chinese Academy of Sciences, Hefei 230031, China.

[2] University of Science and Technology of China, Hefei 230026, China.

[3] Centre for Clean Environment and Energy, Griffith University, Gold Coast Campus, QLD 4222, Australia.

*

These authors contributed equally: Wenyi Li, Ke Li and Yixing Ye. Correspondence and requests for materials should be addressed to C.L. (email: chliang@issp.ac.cn) or to H.Z. (email: zhanghm@issp.ac.cn).

Supplementary Table 1. The comparable results of our work and other recently reported NRR electrocatalysts.

Catalyst	System /Conditions	NH_3 Yield Rate	FE (%)	Detection method	Ref.
**AgNDs**	**0.1 M Na_2SO_4**<br>**(PH =10.5)**	**Ti mesh in three-electrode system**<br>**600.4±23.0 μg h^{-1} mg_{Ag}^{-1}**<br>**(-0.25 V *vs.* RHE)**<br>**Ti plate in two-electrode system**<br>**804.5±30.6 μg h^{-1} mg_{Ag}^{-1}**<br>**(-1.8 V cell voltage)**<br>**O_v-TiO_2/Ti in three-electrode system**<br>**1.27±0.03 μg mL^{-1}**<br>**(-0.25 V *vs.* RHE)**	**16.7±0.9**<br>**(-0.15 V *vs.* RHE)**<br><br>**15.1±0.6**<br>**(-1.5 V cell voltage)**<br><br>**28.9±1.2**<br>**(-0.15 V *vs.* RHE)**	**Indophenol method**	**This work**
**Nanodots Electrocatalysts**					
NiO NDs/G	0.1 M Na_2SO_4	18.6 μg h^{-1} mg^{-1}<br>(-0.7 V *vs.* RHE)	7.8	Indophenol method	1
CuO/RGO	0.1 M Na_2SO_4	1.8×10^{-10} mol s^{-1} cm^{-2}<br>(/)<br>(-0.75 V *vs.* RHE)	3.9	Indophenol method	2
CoO QD/RGO	0.1 M Na_2SO_4	21.5 μg h^{-1} mg^{-1}<br>(-0.6 V *vs.* RHE)	8.3	Indophenol method	3
ZnO QDs/RGO	0.1 M Na_2SO_4	17.7 μg h^{-1} mg^{-1}<br>(-0.65 V *vs.* RHE)	6.4	Indophenol method	4
SnO_2 QDs/RGO	0.1 M Na_2SO_4	25.6 μg h^{-1} mg^{-1}<br>(-0.5 V *vs.* RHE)	7.1	Indophenol method	5
Mo_2C/C	0.5 M Li_2SO_4<br>(PH =2)	11.3 μg h^{-1} mg^{-1}_{Mo2C}<br>(-0.3 V *vs.* RHE)	7.8	Nessler method	6
**Ag-based Electrocatalysts**					
Ag nanosheet	0.1 M HCl	4.62×10^{-11} mol s^{-1} cm^{-2}<br>(/)<br>(-0.60 V *vs.* RHE)	4.8	Indophenol method	7
SA-Ag/NC	0.1 M HCl	270.9 μg h^{-1} $mg_{cat.}^{-1}$<br>69.4 mg h^{-1} mg_{Ag}^{-1}<br>(-0.6 V *vs.* RHE)	21.9<br>(-0.65 V *vs.* RHE)	Indophenol method	8
AgTPs	0.5 M K_2SO_4<br>(pH=3.5)	58.5 μg mg^{-1}_{Ag} h^{-1}<br>(-0.25 V *vs.* RHE)	25	Nessler and Indophenol method	9
BD-Ag/AF	0.1 M Na_2SO_4	2.07×10^{-11} mol s^{-1} cm^{-2}<br>(/)<br>(-0.60 V *vs.* RHE)	9.61<br>(-0.50 V *vs.* RHE)	Indophenol method	10

Ag_3Cu BPNs	0.1 M Na_2SO_4	24.59 μg h^{-1} mg^{-1} (-0.5 V *vs.* RHE)	13.28	Indophenol method	11
Ag-Au@ZIF	$LiCF_3SO_3$ 1% EtOH in THF	10 pmol cm^{-2} s^{-1} (/) (-2.9 V vs. Ag/AgCl)	18±4	Indophenol method	12
**Single-Atom Electrocatalysts**					
SA-Ag/NC	0.1 M HCl	270.9 μg h^{-1} $mg_{cat.}^{-1}$ 69.4 mg h^{-1} mg_{Ag}^{-1} (-0.6 V *vs.* RHE)	21.9 (-0.65 V *vs.* RHE)	Indophenol method	8
AuSAs-NDPCs	0.1 M HCl	2.32 μg h^{-1} cm^{-2} (3.87μg h^{-1} mg^{-1}) (-0.2 V *vs.* RHE)	12.3	Indophenol method	13
Au_1/C_3N_4	0.005 M H_2SO_4	1,305 μg h^{-1} mg^{-1}_{Au} (-0.1 V vs. RHE)	11.1	Indophenol method	14
Ru SAs/N-C	0.05 M H_2SO_4	120.9 μg h^{-1} $mg_{cat.}^{-1}$ (-0.2 V *vs.* RHE)	29.6	Indophenol method	15
$Ru@ZrO_2/NC$	0.1 M HCl	3.665 mg_{NH3} h^{-1} $mg_{Ru.}^{-1}$ (3665 μg h^{-1} $mg_{Ru.}^{-1}$) (-0.21 V *vs.* RHE)	21 (-0.11 V *vs.* RHE)	Indophenol method	16
FeSA-N-C	0.1 M KOH	7.48 μg h^{-1} mg^{-1} (0 V *vs.* RHE)	56.55	Indophenol method	17
SA-Mo/NPC	0.1 M KOH	34.0 ± 3.6 $μg_{NH3}$ h^{-1} $mg_{cat.}^{-1}$ (-0.3 V vs. RHE)	14.6 ±1.6	Nessler method	18
ISAS-Fe/NC	0.1 M PBS	62.9 ± 2.7 μg h^{-1} $mg_{cat.}^{-1}$ (-0.4 V vs. RHE)	18.6 ±0.8	Indophenol method	19
$Fe\text{-}(O\text{-}C_2)_4$	0.1 M KOH	32.1 μg h^{-1} mg^{-1} (-0.1 V *vs.* RHE)	29.3	Indophenol method	20
**Metal Electrocatalysts**					
FePc/C	0.1 M Na_2SO_4	137.95 μg h^{-1} $mg^{-1}_{cat.}$ (-0.3 V vs. RHE)	14.17 (-0.2 V vs. RHE)	Indophenol method	21
Rh_2Sb RNRs/C	0.5 M Na_2SO_4	228.85 ± 12.96 μg h^{-1} mg^{-1}_{Rh} (-0.45 V vs. RHE)	6.32 ±0.28 (-0.30 V vs. RHE)	Indophenol method	22
Bi_4O_{11}/CeO_2	0.1 M HCl (pH = 1)	23.21 μg h^{-1} mg^{-1}_{cat} (-0.2 V *vs.* RHE)	10.16	Indophenol method	23
MXene ($Ti_3C_2T_x$)	0.5 M Li_2SO_4+ 0.1 M HCl (pH=2)	4.72 μg h^{-1} cm^{-2} (2.68 μg h^{-1} mg^{-1}) (-0.1 V *vs.* RHE)	5.78	Nessler method	24
$TiO_2/Ti_3C_2T_x$	0.1 M HCl	32.17 μg h^{-1} mg^{-1}_{cat} (-0.45 V *vs.* RHE)	16.07	Indophenol method	25
$C\text{-}Ti_xO_y/C$	0.1 M $LiClO_4$	14.8 μg h^{-1} mg^{-1}_{cat} (-0.4 V *vs.* RHE)	17.8	Indophenol method	26

Zr-TiO_2	0.1 M KOH	8.90 μg h^{-1} cm^{-2} (8.9 μg h^{-1} mg^{-1}) (-0.45 V *vs.* RHE)	17.3	Indophenol method	27
OVs-rich MoO_2	0.1 M HCl	12.20 μg h^{-1} mg^{-1} (-0.15 V vs. RHE)	8.2	Indophenol method	28
Nb_2O_5/CP	0.1 M HCl	43.6 μg h^{-1} $mg^{-1}_{cat.}$ (-0.55 V vs. RHE)	9.26	Indophenol method	29
Cu-CeO_2-3.9	0.1 M Na_2SO_4 (pH = 6.3)	5.3 × 10^{-10} mol s^{-1} cm^{-1} (13.3 μg h^{-1} $mg_{cat.}^{-1}$) (-0.1 V vs. RHE)	19.1	Indophenol method	30
Mn_3O_4	0.1 M Na_2SO_4	11.6 μg h^{-1} $mg^{-1}_{cat.}$ (-0.8 V *vs.* RHE)	3	Indophenol method	31
Fe/Fe_3O_4	0.1 M PBS (pH = 7.2)	~0.19 μg cm^{-2} h^{-1} (~0.19 μg h^{-1} mg^{-1}) (-0.3 V vs. RHE)	8.29	Indophenol method	32
TiC/C	0.1 M HCl	14.1 μg h^{-1} mg^{-1}_{cat} (-0.5 V *vs.* RHE)	5.8	Indophenol method	33
MoS_2	0.1 M Na_2SO_4	8.08 × 10^{-11} mol s^{-1} cm^{-1} (/) (-0.6 V vs. RHE)	1.17	Indophenol method	34
FeS@MoS_2/CFC	0.1 M Na_2SO_4	8.45 μg cm^{-2} h^{-1} (6.34 μg h^{-1} mg^{-1}) (-0. V *vs.* RHE)	2.96	Nessler method	35
CoS_2/NS-G	0.05 M H_2SO_4	25.0 μg h^{-1} mg^{-1}_{cat} (-0.2 V *vs.* RHE)	25.9 (-0.05 V *vs.* RHE)	Indophenol method	36
LaF_3	0.5 M $LiClO_4$	55.9 μg h^{-1} mg^{-1}_{cat} (-0.45 V *vs.* RHE)	16	Indophenol method	37
PdRu	0.1 M HCl	34.2 μg h^{-1} mg^{-1}_{cat} (-0.2 V *vs.* RHE)	2.4	Indophenol method	38
Pd_3Cu_1	1 M KOH	39.9 μg h^{-1} $mg_{cat.}^{-1}$ (-0.25 V *vs.* RHE)	1.56 (-0.05 V *vs.* RHE)	Nessler method	39
PdCuIr	0.1 M Na_2SO_4	13.43 μg h^{-1} $mg_{cat.}^{-1}$ (-0.3 V vs. RHE)	5.29	Indophenol method	40
**Metal-Free Electrocatalysts**					
CC-450	0.1 M Na_2SO_4 + 0.02 M H_2SO_4	15.8 μg cm^{-2} h^{-1} (/) (-0.3 V *vs.* RHE)	6.92	Indophenol method	41
S-CNS	0.1 M Na_2SO_4	19.07 μg h^{-1} $mg^{-1}_{cat.}$ (-0.3 V *vs.* RHE)	7.47	Indophenol method	42
d-FG	0.1 M Na_2SO_4	9.3 μg h^{-1} mg^{-1}_{cat} (-0.7 V *vs.* RHE)	4.2	Indophenol method	43

FL-BP NSs	0.01M HCl	31.37 μg h^{-1} mg^{-1} (-0.7 V *vs.* RHE)	5.07 (-0.6 V *vs.* RHE)	Indophenol method	44
BG	0.05 M H_2SO_4	9.8 μg cm^{-2} h^{-1} (219.5 μg h^{-1} mg^{-1}) (-0.5 V *vs.* RHE)	10.8	Indophenol method	45
BCN	0.1 M HCl	7.75 μg h^{-1} $mg_{cat.}^{-1}$ (-0.3 V *vs.* RHE)	13.79	Indophenol method	46
NPC	0.05 M H_2SO_4	1.40 mmol g^{-1} h^{-1} (23.8 μg h^{-1} mg^{-1}) (-0.9 V vs. RHE)	1.42	Nessler method	47
VN NPs	0.05M H_2SO_4	3.3×10^{-10} mol s^{-1} cm^{-1} (40.4 μg h^{-1} mg^{-1}) (-0.1 V vs. RHE)	6.0	Nessler method	48
MBN	0.1 M Na_2SO_4	18.2 μg h^{-1} mg^{-1}_{cat} (-0.7 V *vs.* RHE)	5.5	Indophenol method	49
PCN	0.1 M HCl	8.09 μg h^{-1} $mg^{-1}_{cat.}$ (-0.2 V *vs.* RHE)	11.05	Indophenol method	50
B_4C/CPE	0.1 M HCl	26.57 μg h^{-1} $mg^{-1}_{cat.}$ (-0.75 V *vs.* RHE)	15.95	Indophenol method	51

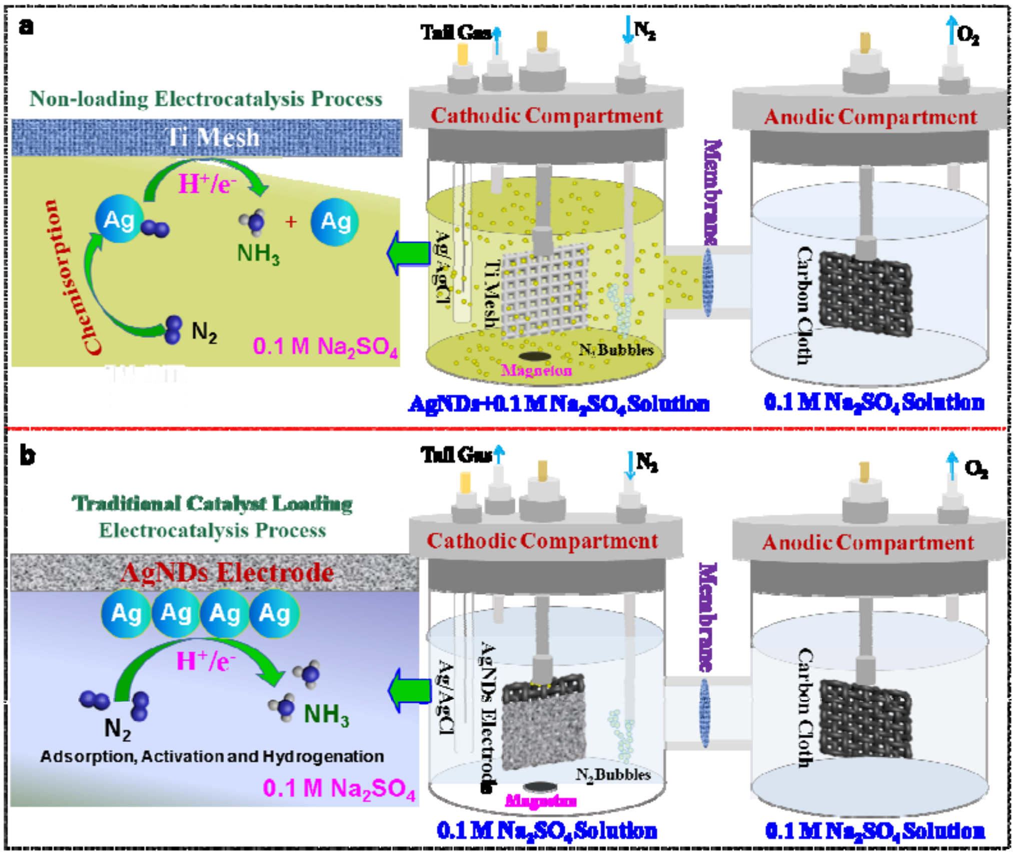

Supplementary Figure 1. Schematic illustration of two NRR electrocatalytic systems. a The non-loading electrocatalysis process using AgNDs catalyst uniformly dispersed in 0.1 M Na_2SO_4 solution (pH=10.5). **b** The traditional catalyst-loading electrocatalysis process in 0.1 M Na_2SO_4 solution (pH=10.5).

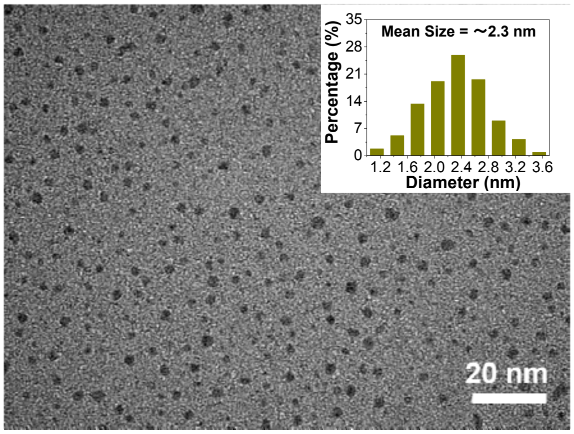

Supplementary Figure 2. TEM characterizations. TEM images of the as-synthesized Ag nanodots by the laser-ablation technique and inset of the corresponding nanodot size distribution curve.

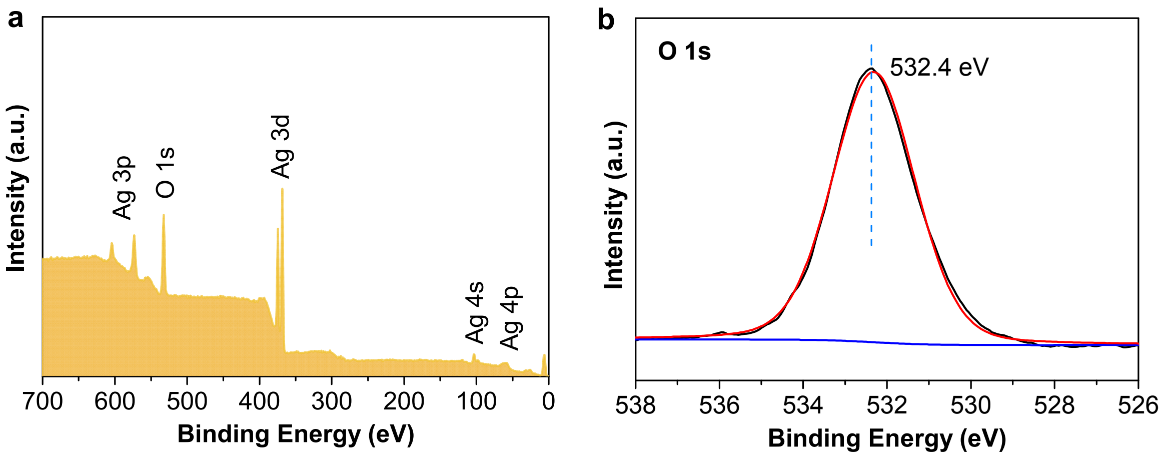

Supplementary Figure 3. XPS characterizations of the AgNDs. a Surface survey XPS spectrum of the AgNDs. **b** High-resolution O 1s XPS spectrum of the AgNDs.

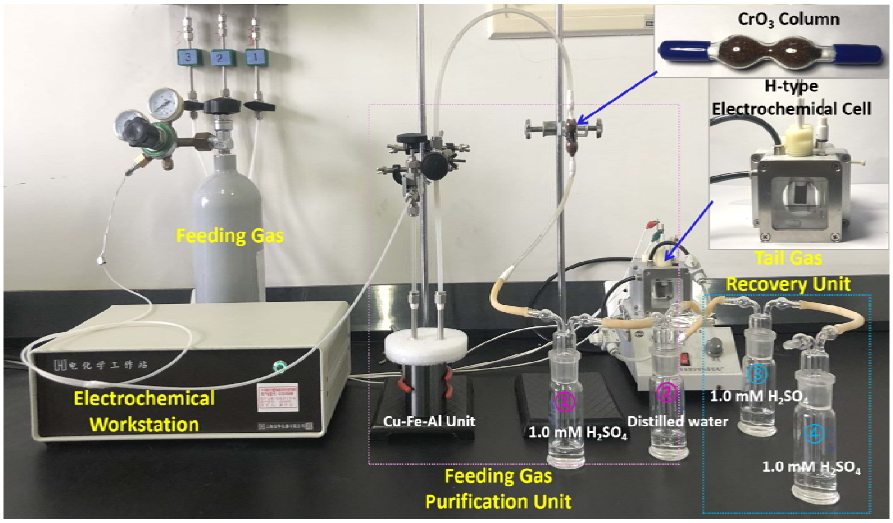

Supplementary Figure 4. Photograph of electrochemical NRR experimental setup.

This experimental set up was established following a similar protocol as the previously reported works to remove the possible interferences of NH_3 and NO_x in the feeding gases.[20,52] Cu-Fe-Al unit was employed to purify the feeding gases. The Cu-Fe-Al catalyst (**Supplementary Figure 5**) was synthesized according to the reported methods.[53-55] Before each experiment, the Cu-Fe-Al catalyst was thermally treated at 300 □ for 2 h under 5% H_2/Ar stream. The treated Cu-Fe-Al catalyst was covered with a stainless-steel vessel which filled up with the mixed ethanol and liquid nitrogen. For complete elimination of NO interference, a CrO_3 catalyst packed column (Dongguan Zhongtian Electronic Technology Co. LTD, China) was also employed in this work. Utilizing this purification system, any NO passed through this purification unit will be converted to water soluble NO_2, and then removed by the H_2SO_4 and distilled water before reaching the electrochemical cell.

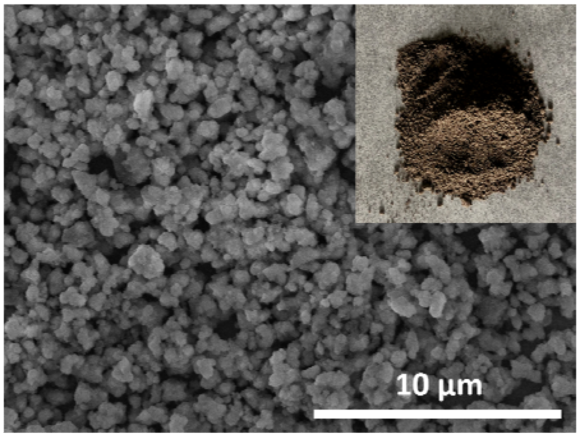

Supplementary Figure 5. SEM image and photograph (inset) of the Cu-Fe-Al catalyst.

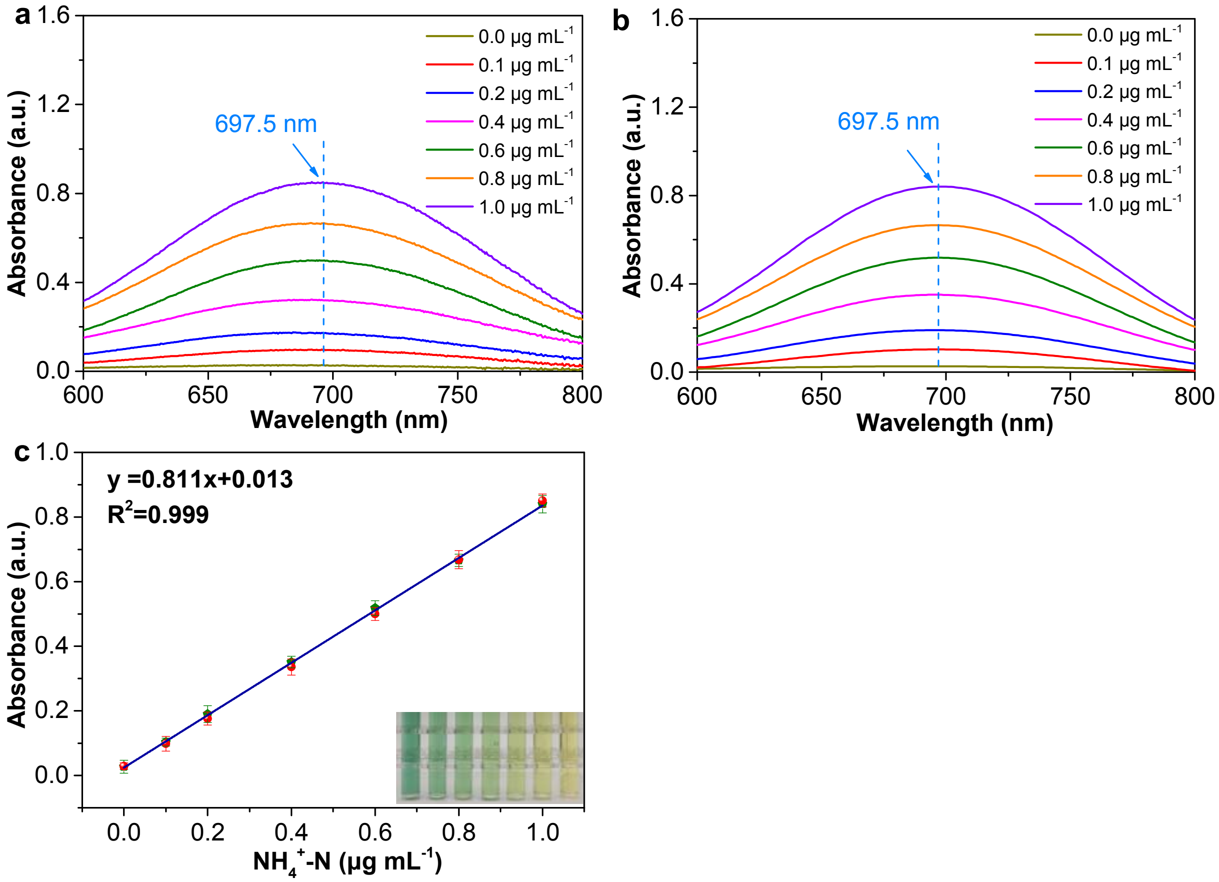

Supplementary Figure 6. Determination of ammonia. UV-Vis absorption spectra of the indophenol blue indicator with various concentrations of NH_4^+-N (0, 0.1, 0.2, 0.4, 0.8, 1.0 μg mL^{-1}) after incubating for 1 h at room temperature, **a** in 0.1 M Na_2SO_4 electrolyte (pH=10.5) and **b** in 0.1 M Na_2SO_4 electrolyte (pH=10.5) with AgNDs incorporation. **c** The calibration curve used for calculation of NH_4^+-N concentration (red sphere: in 0.1 M Na_2SO_4 electrolyte; green sphere: in 0.1 M Na_2SO_4 electrolyte with AgNDs incorporation). The error bars correspond to the standard deviations of three independent measurements.

The above results indicate that the influence of AgNDs introduction in 0.1 M Na_2SO_4 electrolyte (pH=10.5) is ignorable for the detection of NH_3 product by the indophenol blue method.

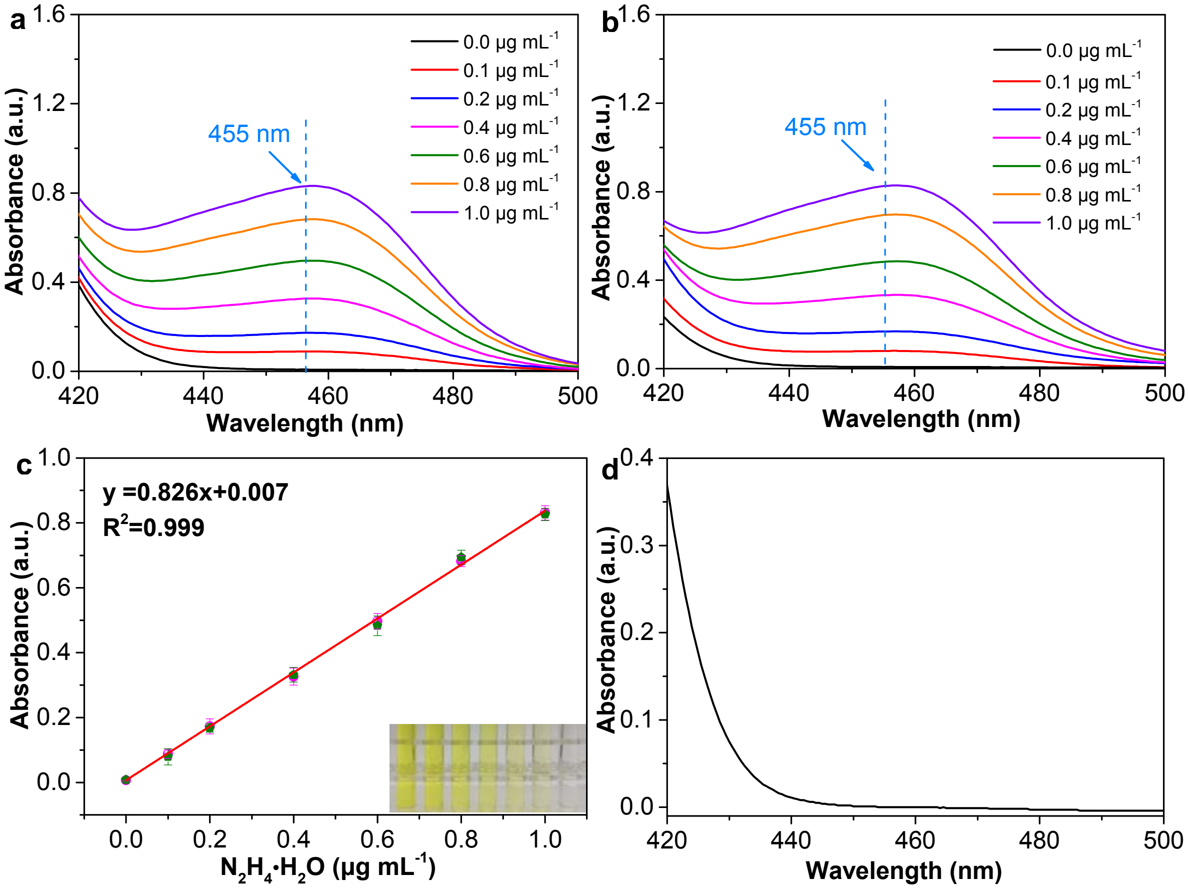

Supplementary Figure 7. Determination of hydrazine. UV-Vis absorption spectra with various concentrations of $N_2H_4{\cdot}H_2O$ (0, 0.1, 0.2, 0.4, 0.6, 0.8, 1.0 μg mL^{-1}) after incubated for 20 min at room temperature, **a** in 0.1 M Na_2SO_4 electrolyte (pH=10.5) and **b** in 0.1 M Na_2SO_4 electrolyte (pH=10.5) with AgNDs incorporation. **c** The calibration curve used for calculation of $N_2H_4{\cdot}H_2O$ concentrations (Magenta sphere: in 0.1 M Na_2SO_4 electrolyte (pH=10.5); green sphere: in 0.1 M Na_2SO_4 electrolyte (pH=10.5) with AgNDs incorporation. The error bars correspond to the standard deviations of three independent measurements. **d** UV-Vis absorption spectra of samples after NRR measurement at -0.25 V *vs.* RHE in 0.1 M Na_2SO_4 electrolyte (pH=10.5) with AgNDs incorporation.

The above results indicate that the influence of AgNDs introduction in 0.1 M Na_2SO_4 electrolyte (pH=10.5) is ignorable for the detection of $N_2H_4{\cdot}H_2O$ by the Watt and Chrisp method.

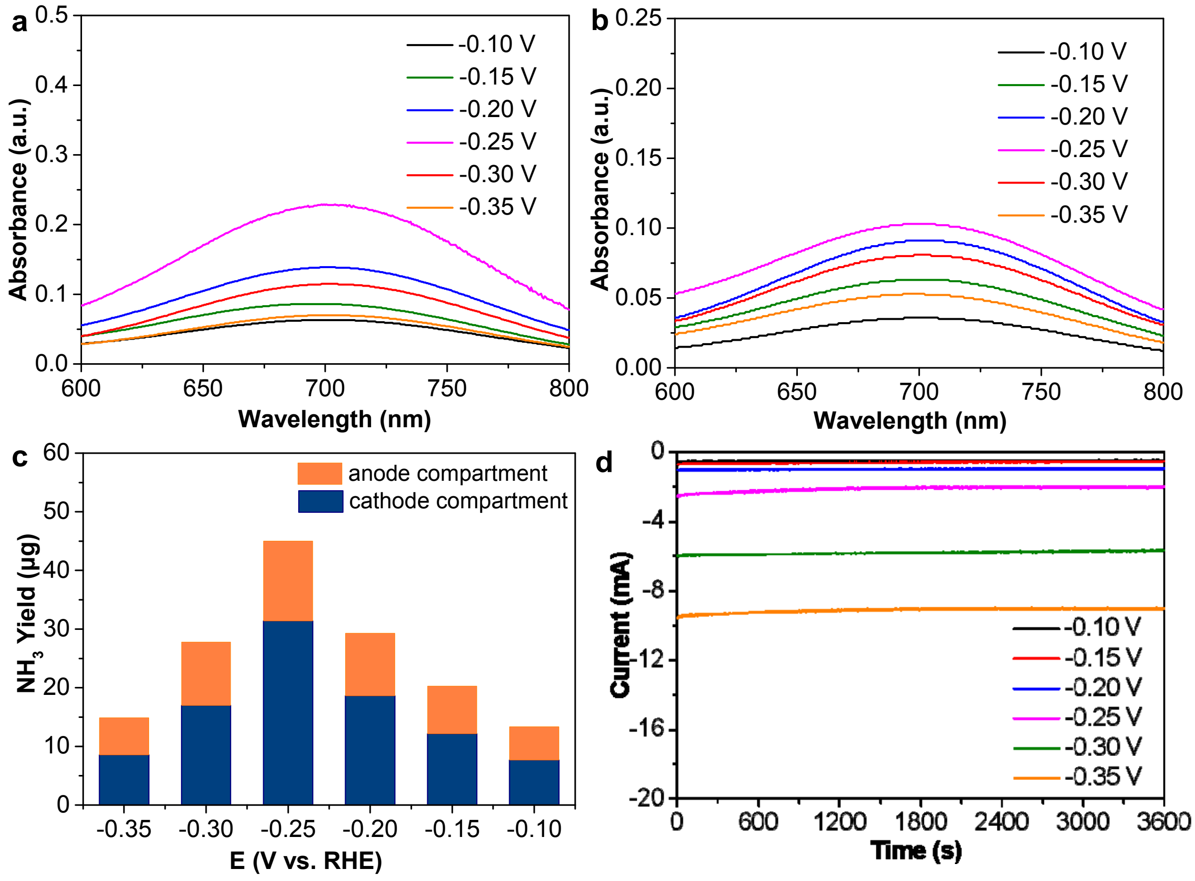

Supplementary Figure 8. The characterizations of the NH_3 yield of AgNDs catalyst on the applied potential. UV-Vis absorption spectra of the collected samples from **a** cathodic compartment and **b** anodic compartment after NRR for 1 h in N_2-saturated 0.1 M Na_2SO_4 electrolyte (pH=10.5) at different potentials. **c** Total NH_3 yield using AgNDs in N_2-saturated 0.1 M Na_2SO_4 electrolyte (pH=10.5) at different potentials. **d** Time-dependent current curves of AgNDs at different potentials in N_2-saturated 0.1 M Na_2SO_4 electrolyte (pH=10.5).

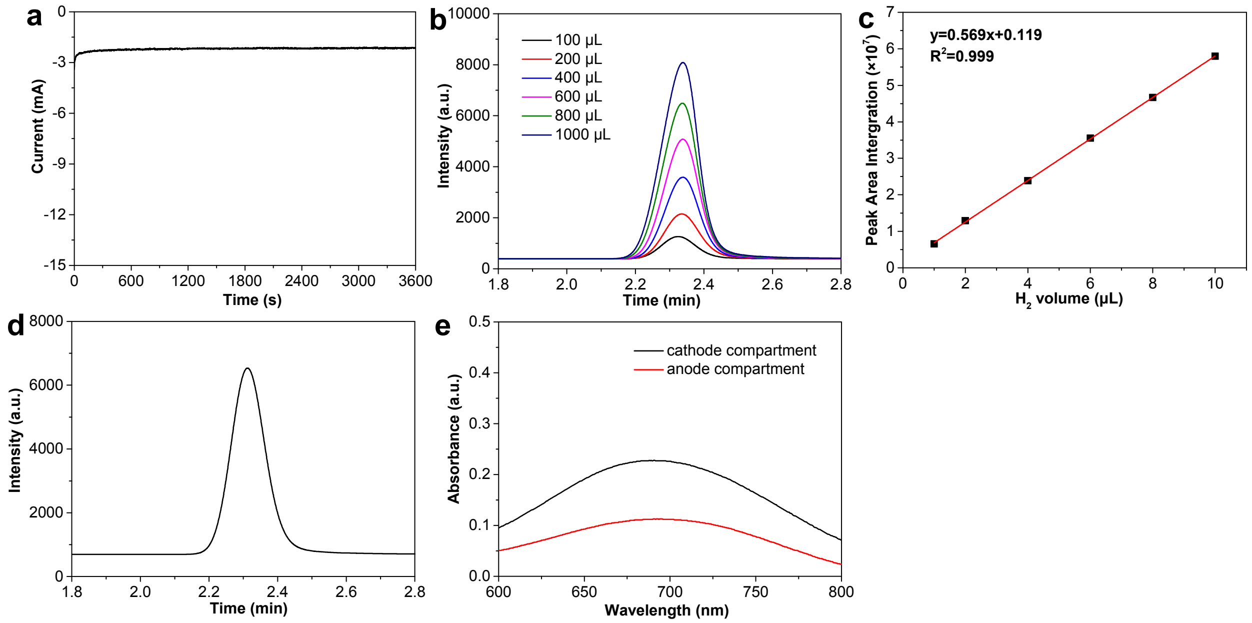

Supplementary Figure 9. The quantification of the produced H_2. a The time-dependent current curve of NRR measurement at -0.25 V *vs.* RHE in 0.1 M Na_2SO_4 electrolyte (pH=10.5) with AgNDs incorporation. **b** Chromatograms of H_2 standards (100, 200, 400, 600, 800, 1000 μL). **c** The calibration curve used for calculation of H_2 volume. **d** The chromatogram of the yielded H_2 after 1 h NRR measurement at -0.25 V *vs.* RHE in 0.1 M Na_2SO_4 electrolyte (pH=10.5) with AgNDs incorporation. **e** The UV-Vis absorption spectra of yielded NH_3 after 1 h NRR measurement at -0.25 V *vs.* RHE in 0.1 M Na_2SO_4 electrolyte (pH=10.5) with AgNDs incorporation.

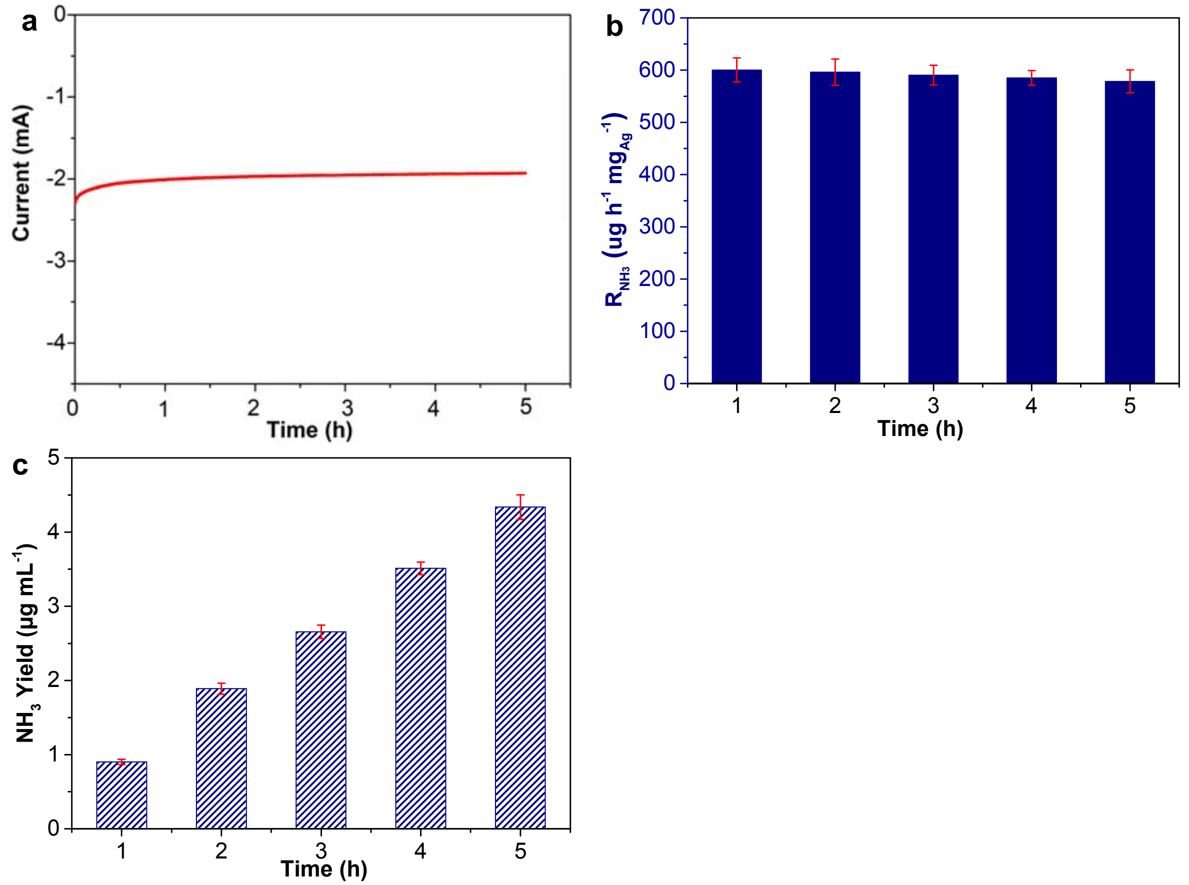

Supplementary Figure 10. Durability test. a Durability test of AgNDs catalyst at -0.25 V (*vs.* RHE) for 5 h NRR and the corresponding **b** R_{NH3} and **c** NH_3 yield measured every 1 h during 5 h NRR. The error bars correspond to the standard deviations of three independent measurements.

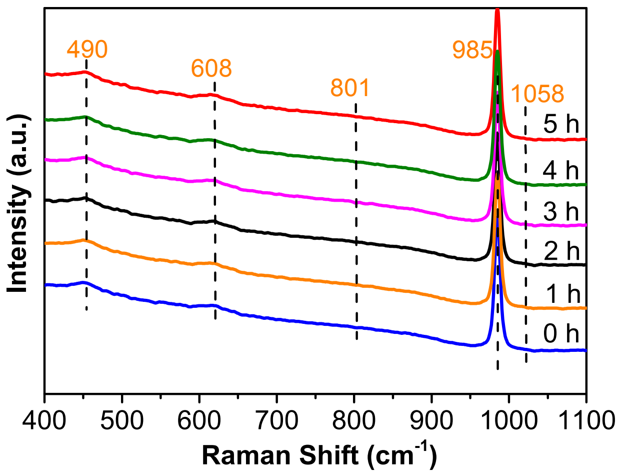

Supplementary Figure 11. *In-situ* time-dependence Raman spectra of AgNDs during durability measurement of NRR.

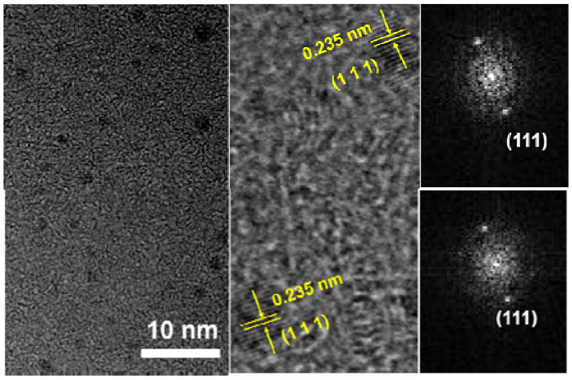

Supplementary Figure 12. TEM characterizations after 5 h NRR measurement. TEM and HRTEM images (inset of corresponding FFT patterns) of AgNDs after 5 h NRR measurement.

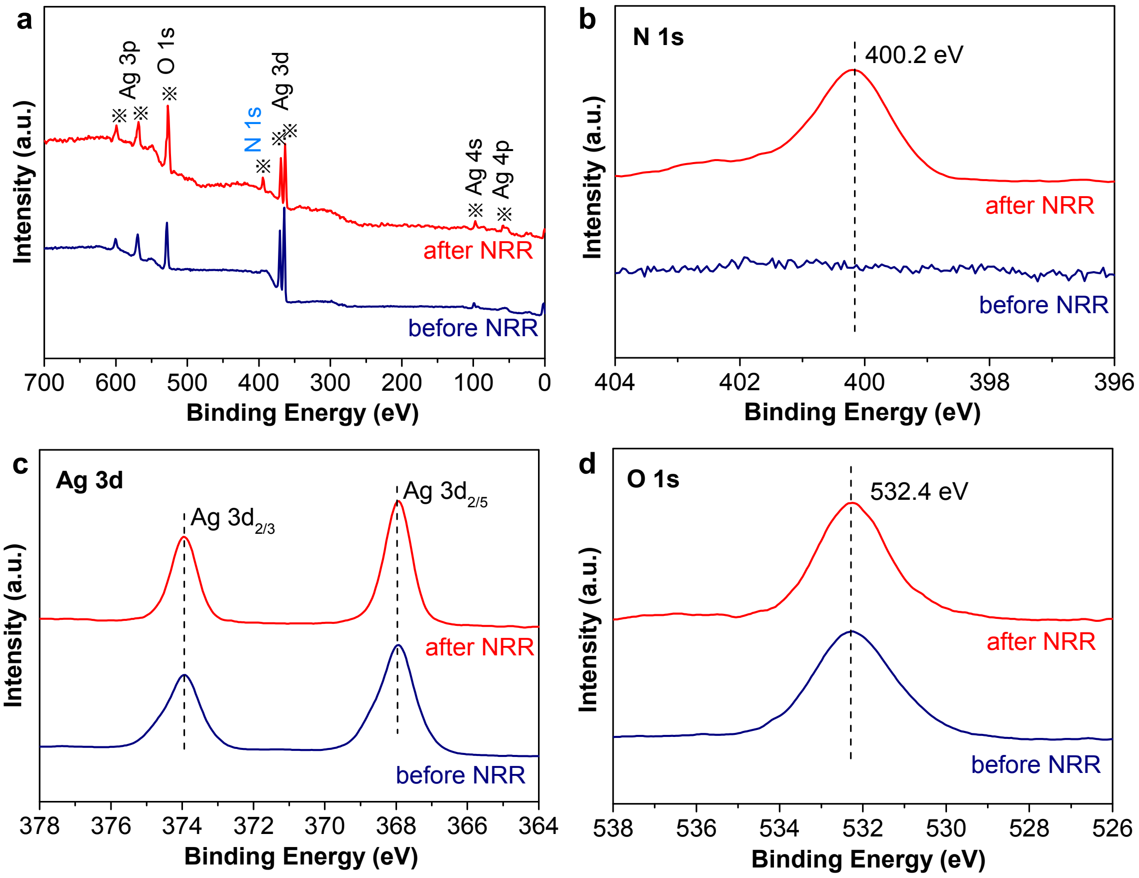

Supplementary Figure 13. XPS characterizations of the AgNDs after 5 h NRR measurement. a Surface survey XPS spectrum, **b** High-resolution N 1s XPS spectrum, **c** High-resolution Ag 3d XPS spectrum, **d** High-resolution O 1s XPS spectrum of the AgNDs before and after 5 h NRR measurement.

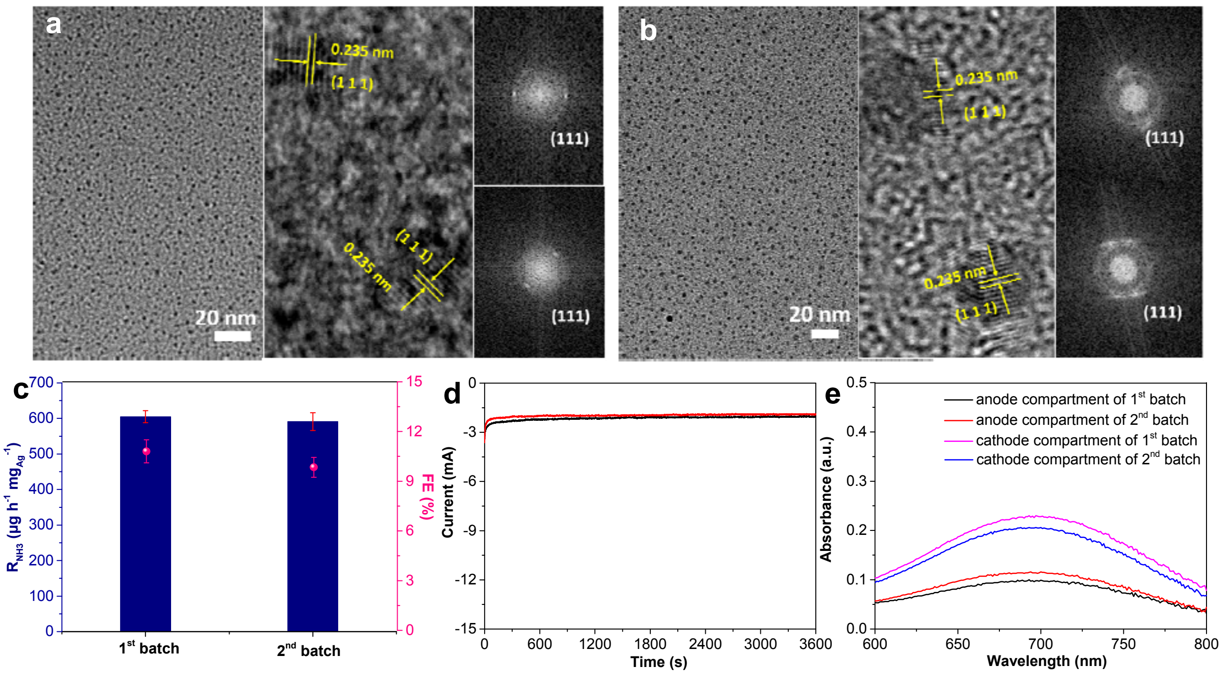

Supplementary Figure 14. Reproducibility test. TEM and HRTEM images (insets of corresponding FFT patterns) of **a** 1st batch and **b** 2nd batch of AgNDs. **c** NH_3 yield rate of two batches of AgNDs at -0.25 V (*vs.* RHE) in N_2-saturated 0.1 M Na_2SO_4 electrolyte (pH=10.5) for 1 h NRR in the non-loading electrocatalysis system. The error bars correspond to the standard deviations of three independent measurements. **d** The corresponding time-dependent current curves. **e** The corresponding UV-Vis absorption spectra of the collected solution samples.

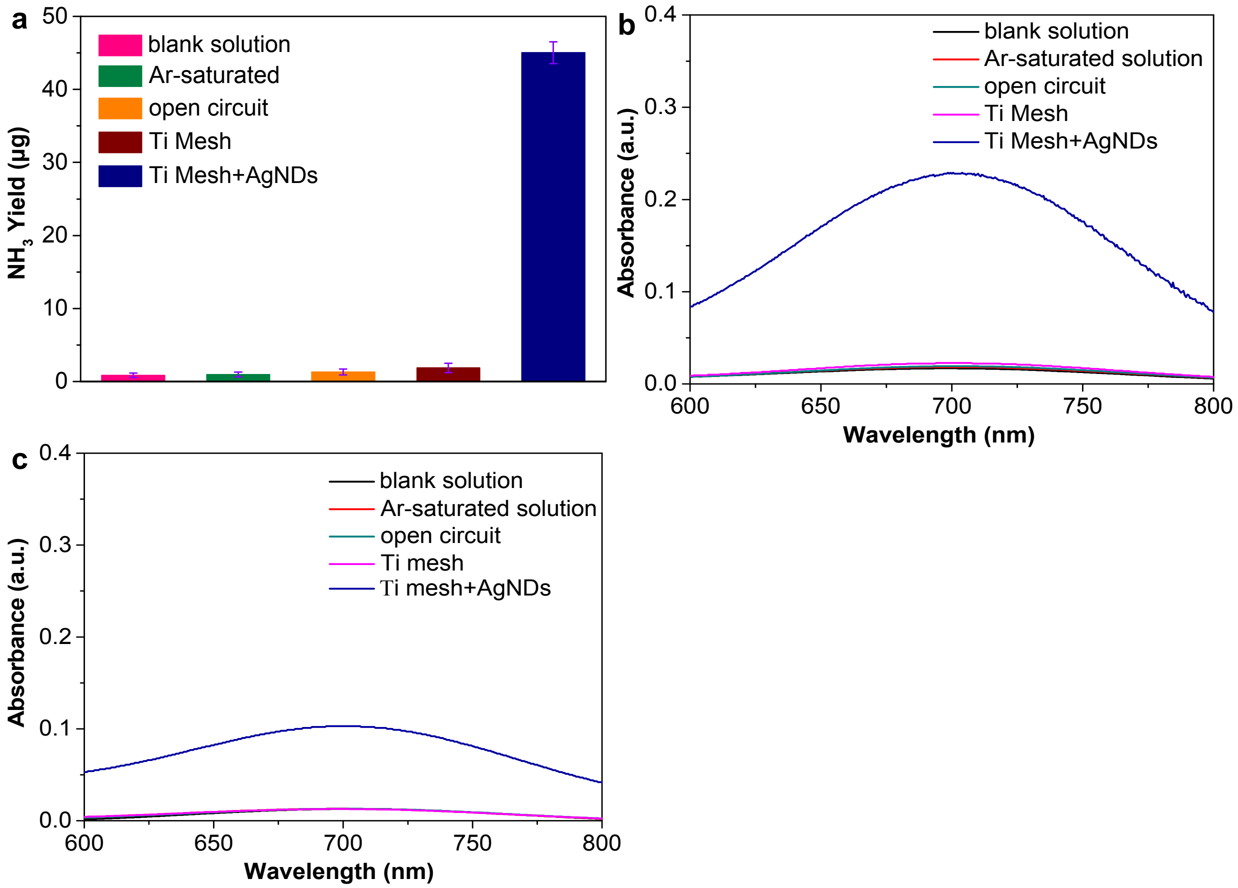

Supplementary Figure 15. NH_3 quantification of the control experiments. a NH_3 yield under different control experiment conditions. The error bars correspond to the standard deviations of three independent measurements. UV-Vis absorption spectra of the corresponding collected solution samples from **b** cathodic compartment and **c** anodic compartment.

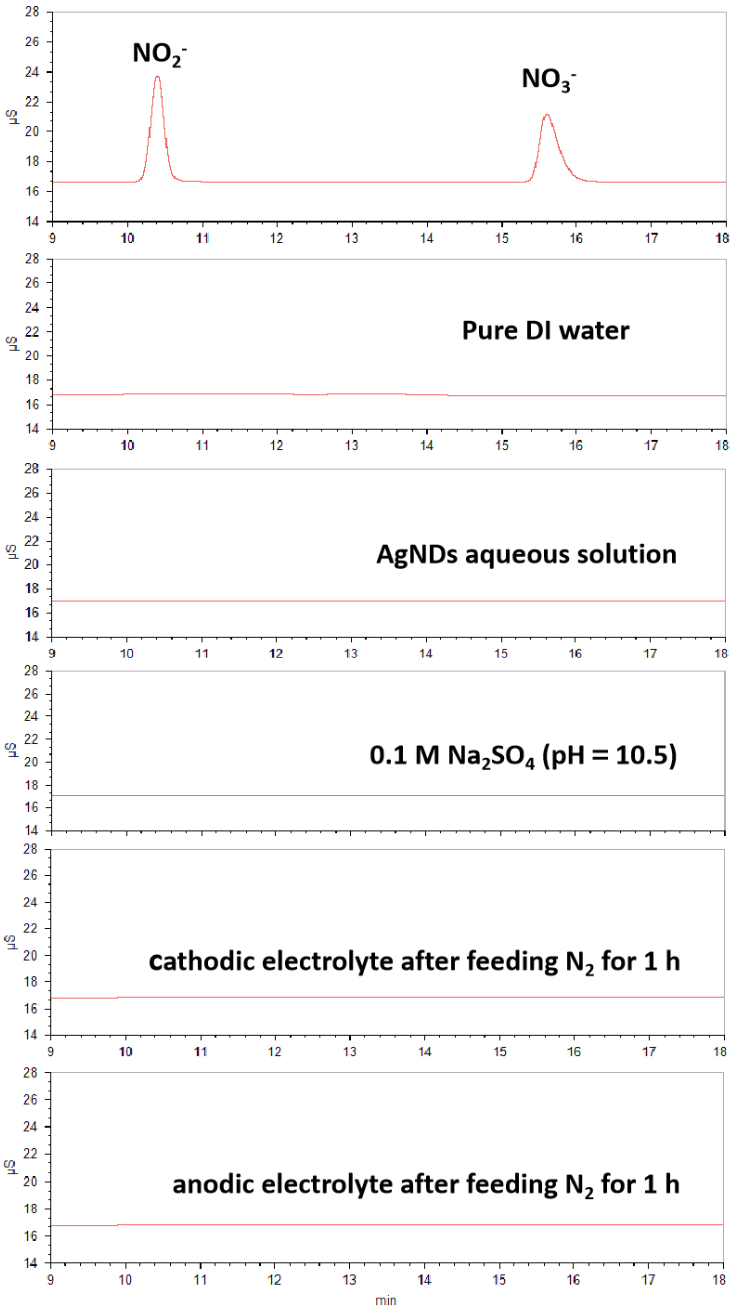

Supplementary Figure 16. Chromatograms of the standard solution (5.0 ppm NO_2^- and 5.0 ppm NO_3^-) and the collected samples under varoius conditions.

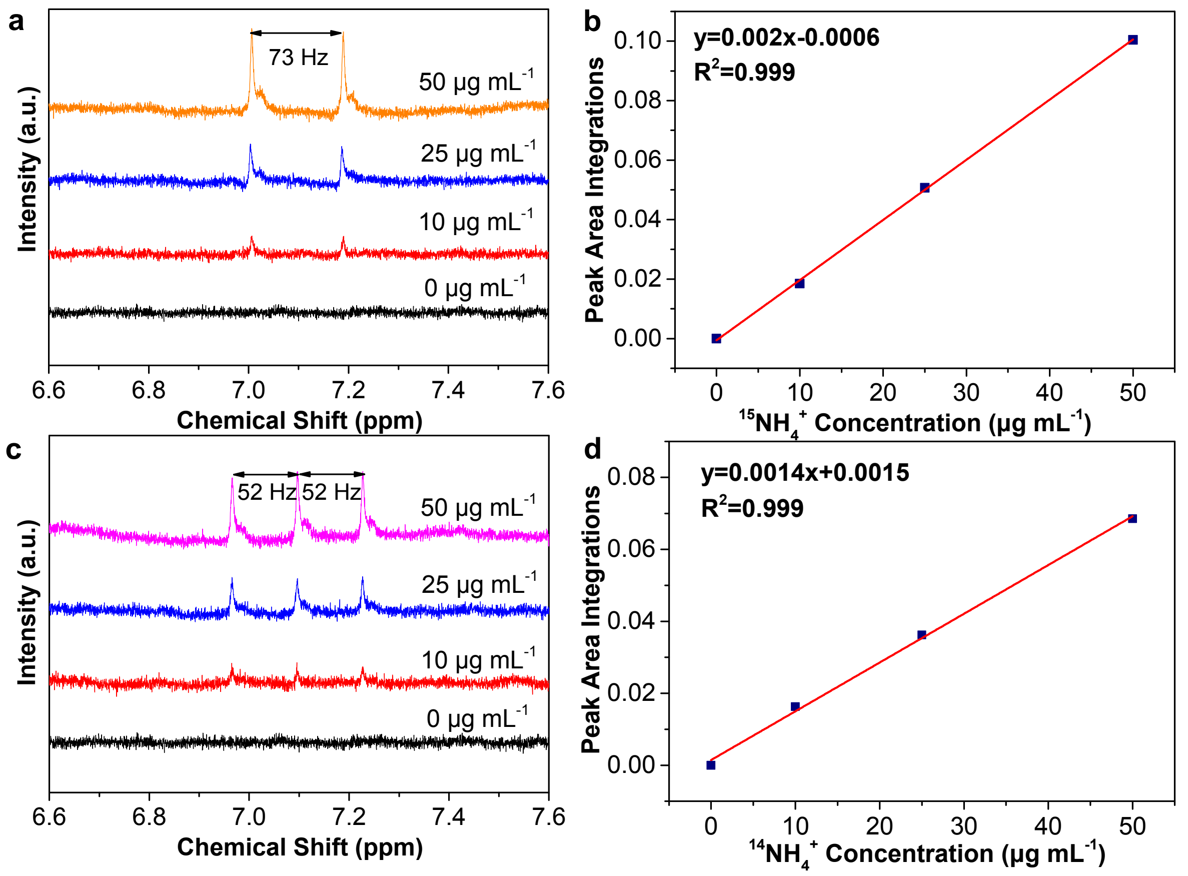

Supplementary Figure 17. Isotopic labelling quantification experiments. a, c ^{1}H NMR spectra of the $^{15}NH_4^+$ and $^{14}NH_4^+$ standards with different concentrations. **b, d** Corresponding $^{15}NH_4^+$ and $^{14}NH_4^+$ calibration curves constructed by plotting the integrated ^{1}H NMR signal (7.00 ppm for $^{15}NH_4^+$ and 6.97 ppm for $^{14}NH_4^+$) against the standard concentration.

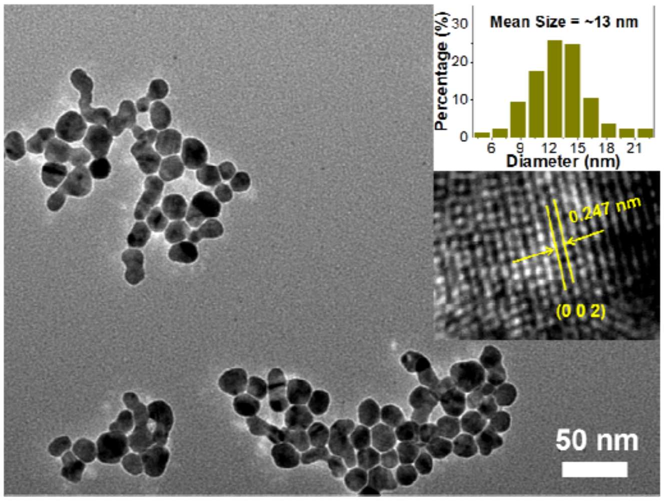

Supplementary Figure 18. TEM image of the as-synthesized Ag_2ONPs. (Inset of the corresponding Ag_2O nanoparticles size distribution curve and HRTEM image).

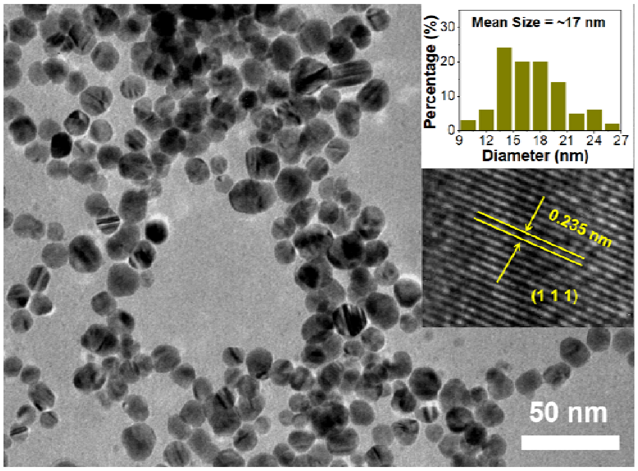

Supplementary Figure 19. TEM image of the as-synthesized AgNPs. (Inset of the corresponding Ag nanoparticles size distribution curve and HRTEM image).

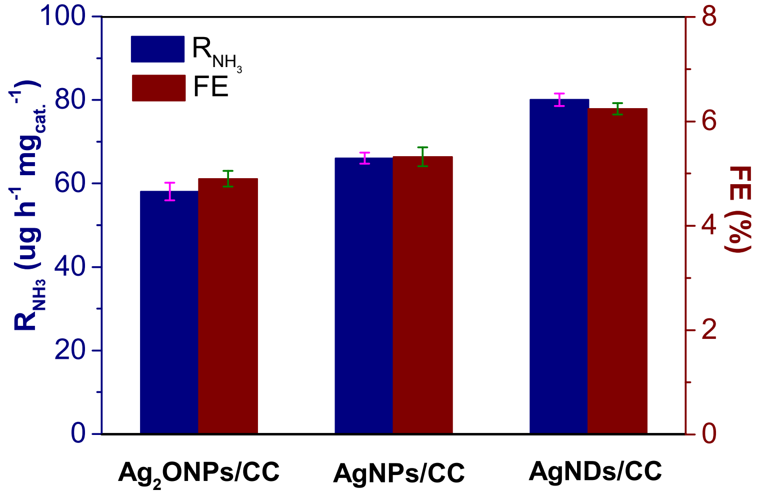

Supplementary Figure 20. Contrast experiments. NH_3 yield rate and faradaic efficiency of Ag_2ONPs/CC, AgNPs/CC and AgNDs/CC at -0.25 V (*vs.* RHE) in N_2-saturated 0.1 M Na_2SO_4 electrolyte (pH=10.5) for 1 h NRR. The error bars correspond to the standard deviations of three independent measurements.

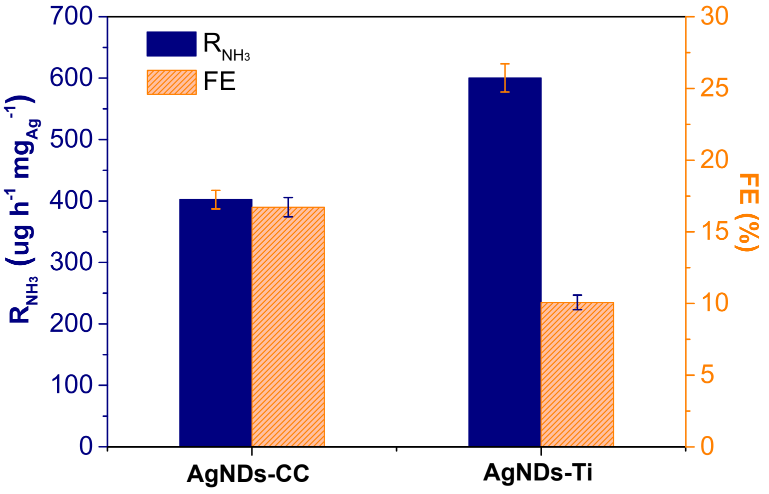

Supplementary Figure 21. Contrast experiments. NH_3 yield rate and faradaic efficiency of AgNDs catalyst using Ti mesh and carbon cloth (CC) as the current collectors in the non-loading electrocatalysis system. The error bars correspond to the standard deviations of three independent measurements.

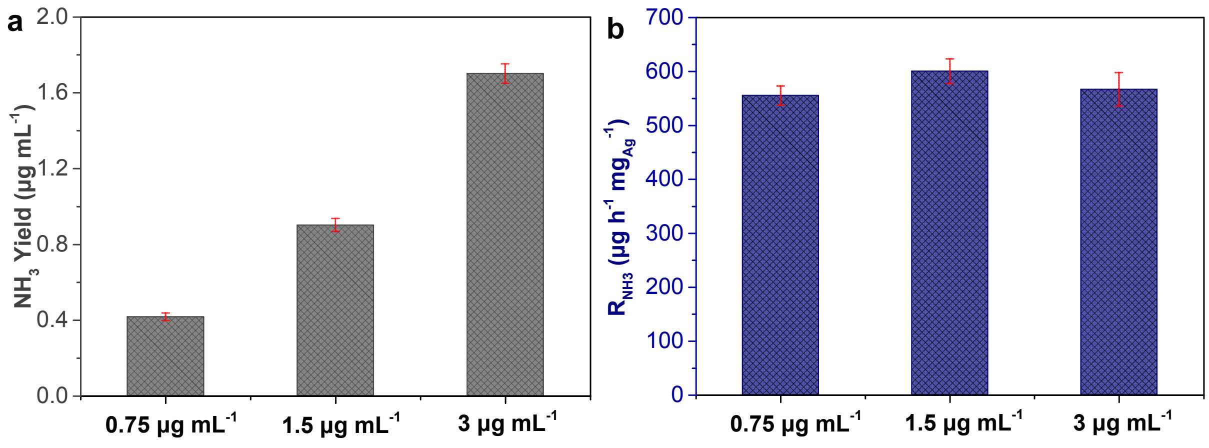

Supplementary Figure 22. The influence of AgNDs concentration on the NH_3 yield. a NH_3 yield and **b** NH_3 yield rate of different concentrations of AgNDs at -0.25 V (*vs.* RHE) in N_2-saturated 0.1 M Na_2SO_4 electrolyte (pH=10.5) for 1 h NRR in the non-loading electrocatalysis system. The error bars correspond to the standard deviations of three independent measurements.

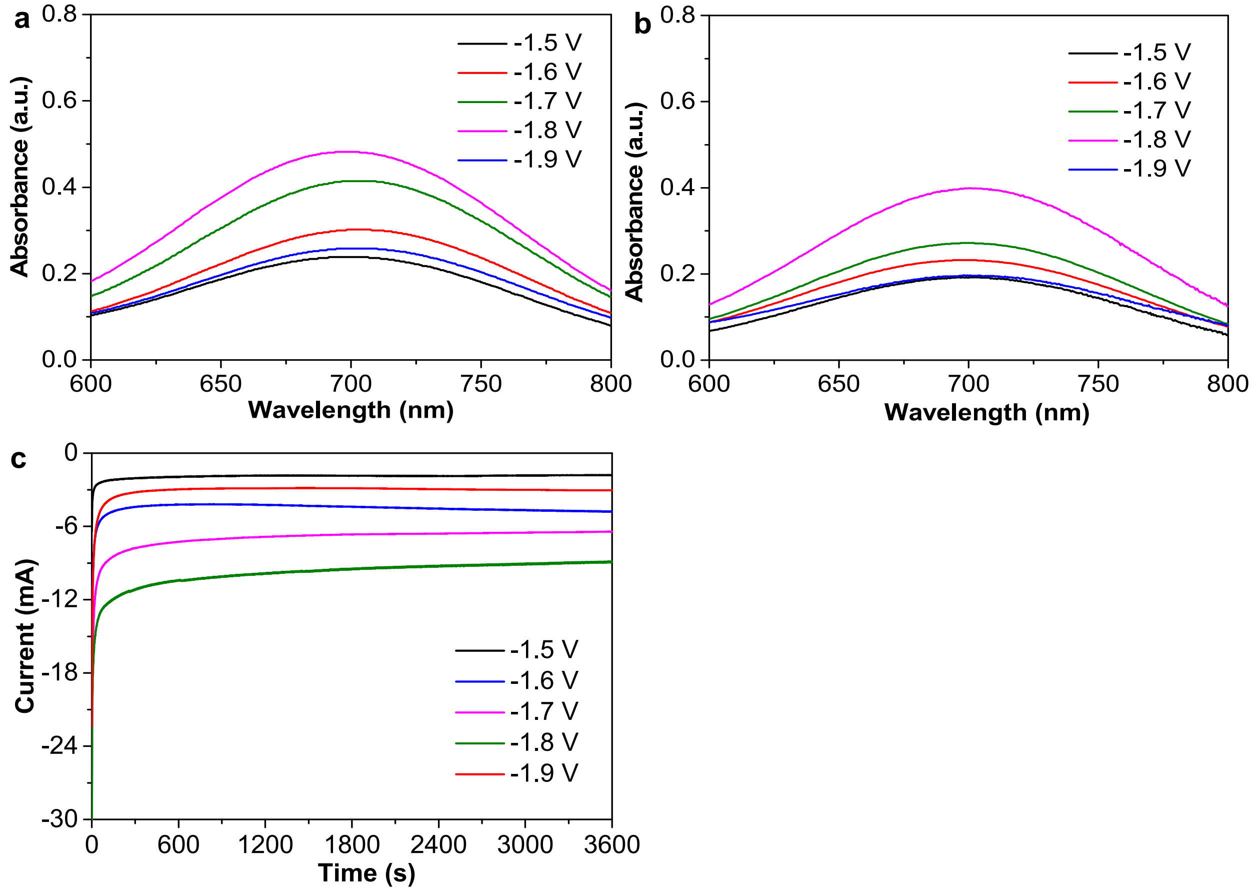

Supplementary Figure 23. The characterizations of the NH_3 yield of AgNDs catalyst in Ti plate-based two-electrode configured flow-type electrochemical reactor. UV-Vis absorption spectra of the collected samples from **a** cathodic compartment and **b** anodic compartment after NRR for 1 h in N_2-saturated 0.1 M Na_2SO_4 electrolyte (pH=10.5) at different cell voltages. **c** Time-dependent current curves of AgNDs at different cell voltages in N_2-saturated 0.1 M Na_2SO_4 electrolyte (pH=10.5).

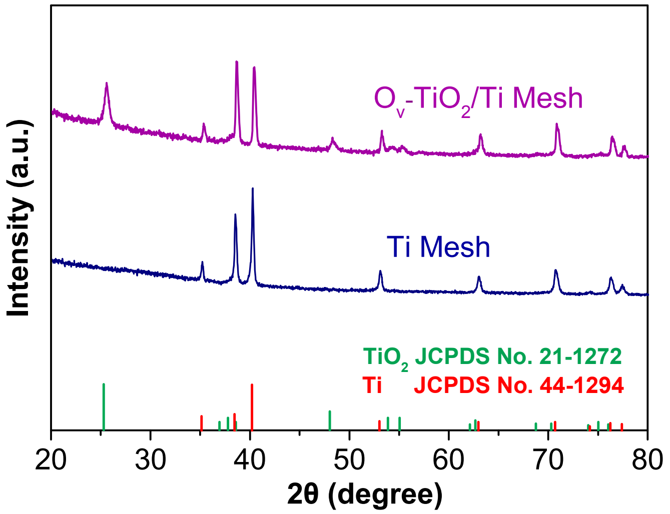

Supplementary Figure 24. XRD patterns of the pristine Ti mesh and O_v-TiO_2 /Ti mesh.

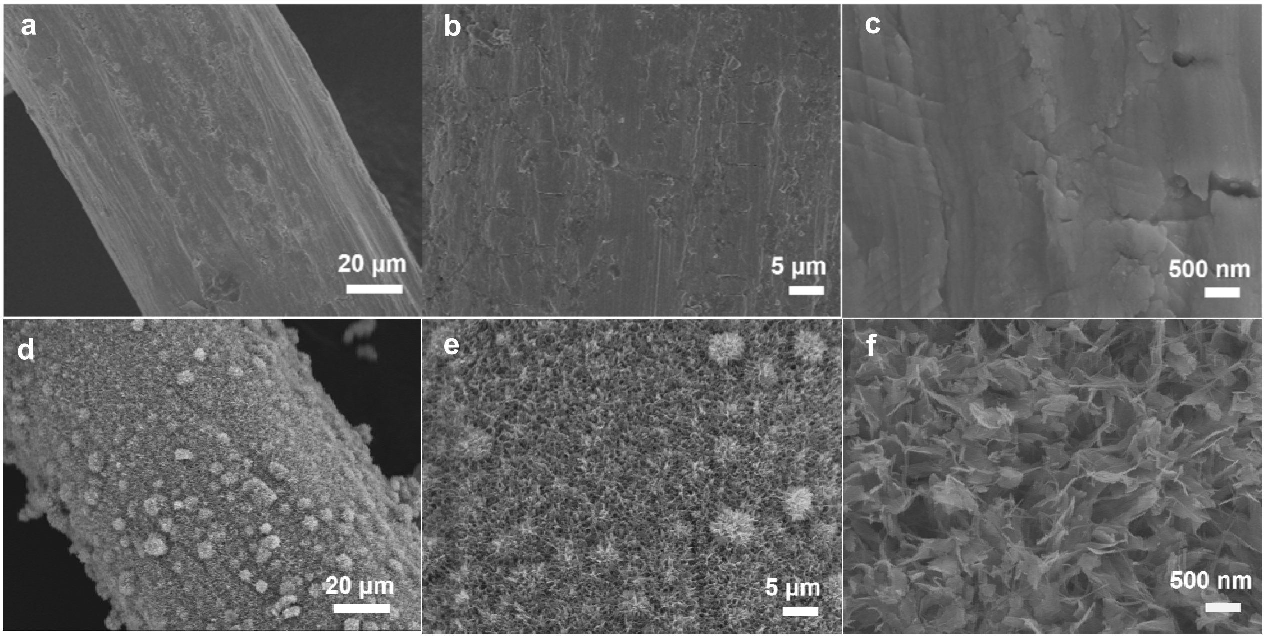

Supplementary Figure 25. SEM characterizations. SEM images of **a-c** pristine Ti mesh and **d-f** O_v-TiO_2/Ti mesh with different magnifications.

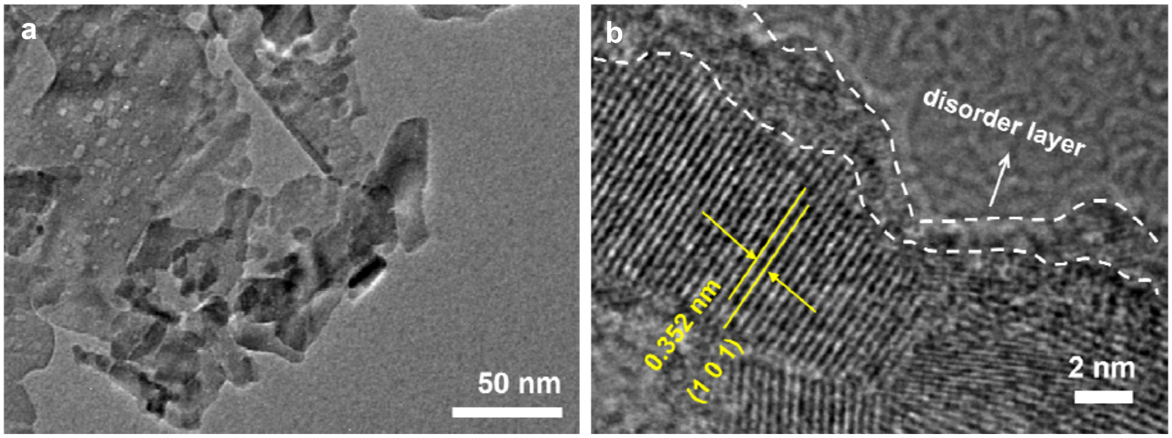

Supplementary Figure 26. TEM characterizations. a TEM and **b** corresponding HRTEM images of O_v-TiO_2/Ti mesh.

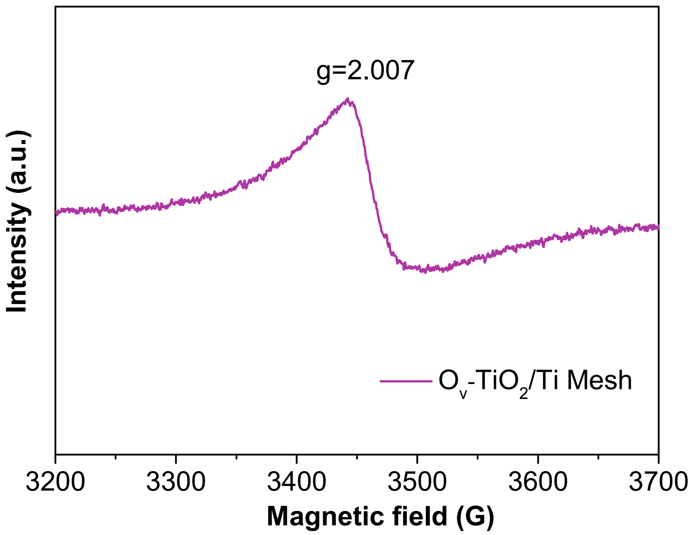

Supplementary Figure 27. EPR spectra of O_v-TiO_2/Ti mesh.

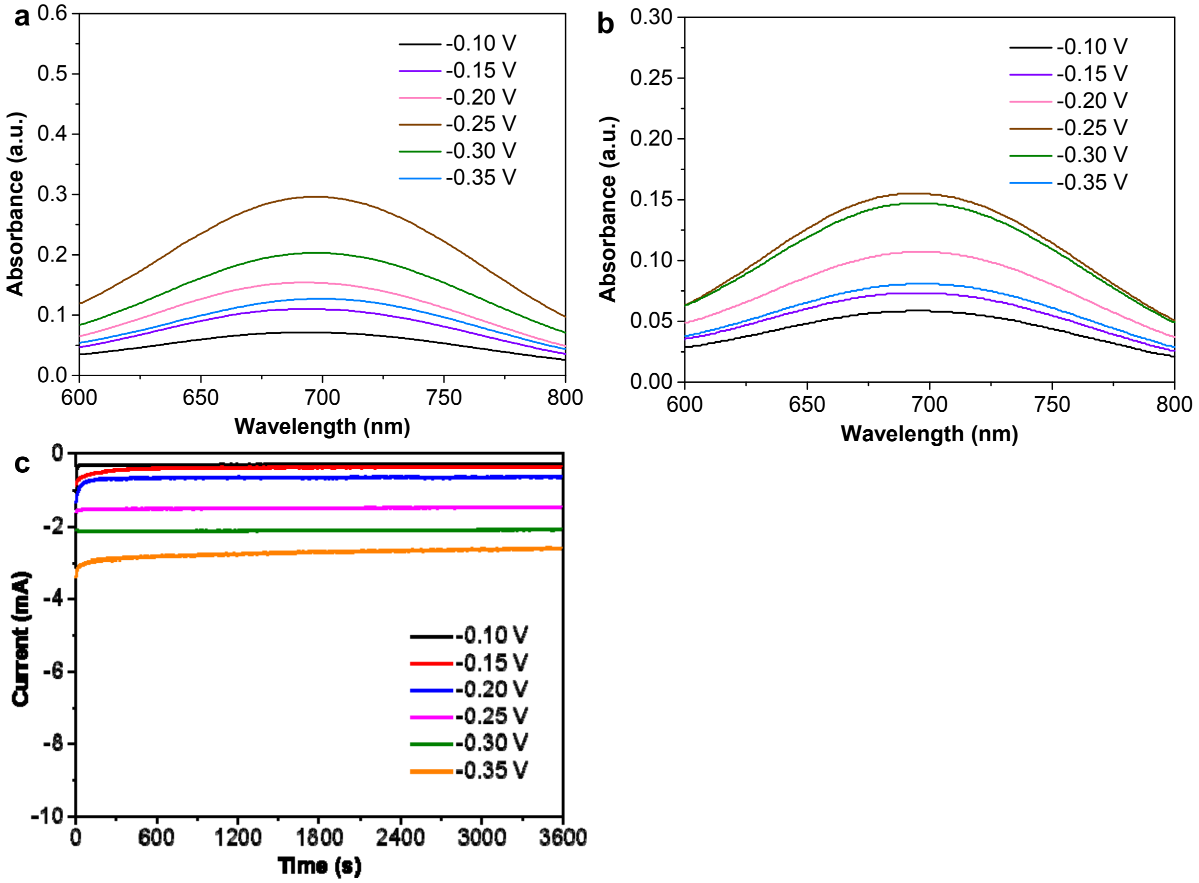

Supplementary Figure 28. The characterizations of the NH_3 yield of AgNDs catalyst using O_v-TiO_2/Ti current collector. UV-Vis absorption spectra of the collected samples from **a** cathodic compartment and **b** anodic compartment after NRR for 1 h at different potentials using O_v-TiO_2/Ti current collector and AgNDs in N_2-saturated 0.1 M Na_2SO_4 electrolyte (pH=10.5). **c** Time-dependent current curves of AgNDs at different potentials in N_2-saturated 0.1 M Na_2SO_4 electrolyte (pH=10.5) using O_v-TiO_2/Ti current collector.

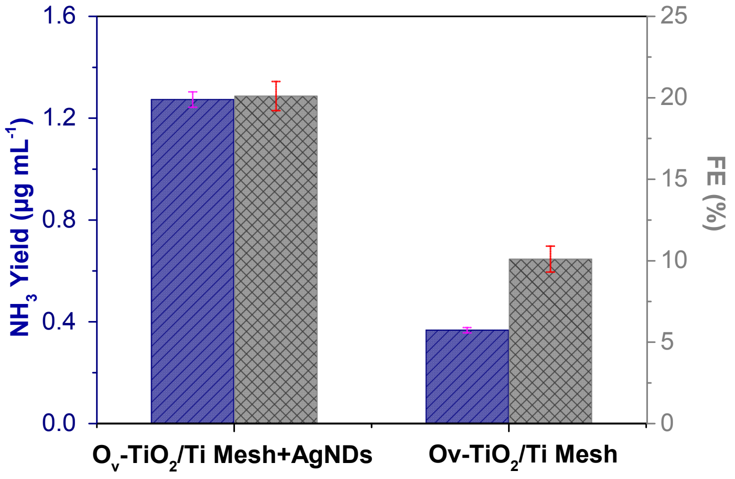

Supplementary Figure 29. Contrast experiments. Comparison of NH_3 yield and FE for O_v-TiO_2/Ti with and without AgNDs incorporation in N_2-saturated 0.1 M Na_2SO_4 electrolyte (pH=10.5) at -0.25 V *vs.* RHE. The error bars correspond to the standard deviations of three independent measurements.

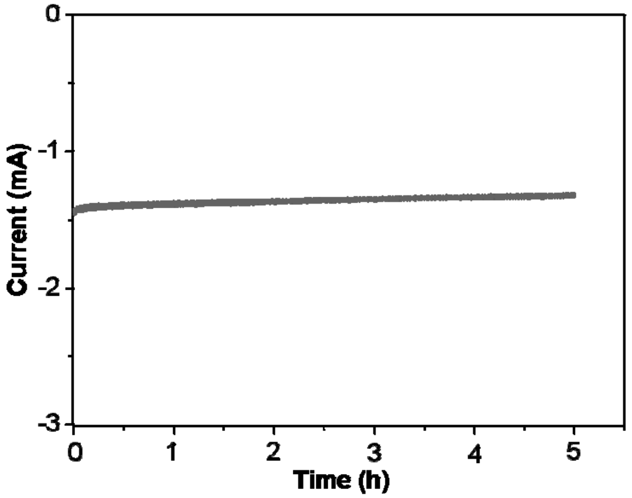

Supplementary Figure 30. Durability test. Time-dependent current curve of O_v-TiO_2/Ti with AgNDs incorporation in N_2-saturated 0.1 M Na_2SO_4 electrolyte (pH=10.5) at -0.25 V *vs*. RHE for 5 h NRR.

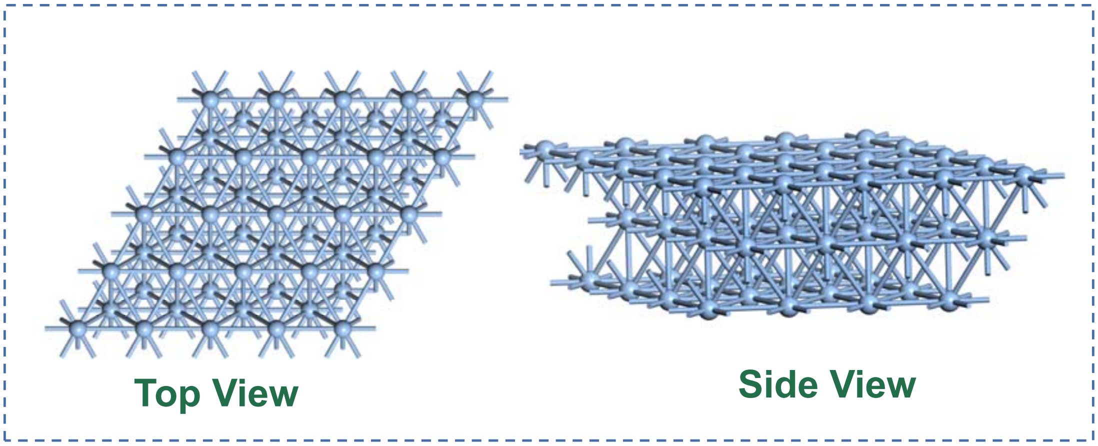

Supplementary Figure 31. The optimized geometric structure model of Ag(111) plane.

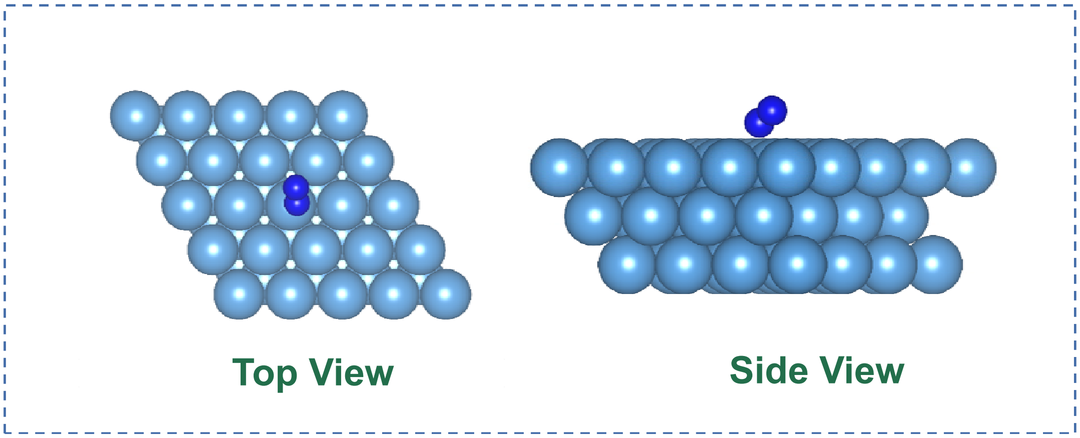

Supplementary Figure 32. Top view and side view of relaxed N_2 adsorption configurations on Ag(111) plane. (Ag: cyan, N: dark blue)

Supplementary References

1. Chu, K. et al. NiO nanodots on graphene for efficient electrochemical N_2 reduction to NH_3. *ACS Appl. Energy Mater.* **2**, 2288-2295 (2019).
2. Wang, F. et al. CuO/Graphene nanocomposite for nitrogen reduction reaction. *ChemCatChem* **11**, 1441-1447 (2019).
3. Chu, K. et al. Efficient electrocatalytic N_2 reduction on CoO quantum dots. *J. Mater. Chem. A* **7**, 4389-4394 (2019).
4. Liu, Y. P. et al. ZnO quantum dots coupled with graphene toward electrocatalytic N_2 reduction: experimental and DFT investigations. *Chem. Eur. J.* **25**, 11933-11939 (2019).
5. Chu, K. et al. Electronically coupled SnO_2 quantum dots and graphene for efficient nitrogen reduction reaction. *ACS Appl. Mater. Interfaces* **11**. 31806-31815 (2019).
6. Cheng, H. et al. Molybdenum carbide nanodots enable efficient electrocatalytic nitrogen fixation under ambient conditions. *Adv. Mater.* **30**, 1803694 (2018).
7. Sun, X. et al. Ag nanosheets for efficient electrocatalytic N_2 fixation to NH_3 under ambient conditions. *Chem. Commun.* **54**, 11427-11430 (2018).
8. Chen, Y. et al. Highly productive electrosynthesis of ammonia by admolecule-targeting single Ag sites. *ACS Nano* **14**, 6938-6946 (2020).
9. Gao, W. Y. et al. Morphology-dependent electrocatalytic nitrogen reduction on Ag triangular nanoplates. *Chem. Commun.* **55**, 10705-10708 (2019).
10. Ji, L. et al. Nanostructured bromide-derived Ag film: an efficient electrocatalyst for N_2 reduction to NH_3 under ambient conditions. *Inorg. Chem.* **57**, 14692-14697 (2018).
11. Yu, H., *et al.* Bimetallic Ag_3Cu porous networks for ambient electrolysis of nitrogen to ammonia. *J. Mater. Chem. A* **7**, 12526-12531 (2019).
12. Lee, H.K., *et al.* Favoring the unfavored: Selective electrochemical nitrogen fixation using a reticular chemistry approach. *Sci. Adv.* **4**, eaar3208 (2018).
13. Qin, Q., Heil, T., Antonietti, M. & Oschatz, M. Single-site gold catalysts on hierarchical N-doped porous noble carbon for enhanced electrochemical reduction of nitrogen. *Small Methods* **2**, 1800202 (2018).
14. Wang, X., *et al.* Atomically dispersed Au_1 catalyst towards efficient electrochemical synthesis of ammonia. *Sci. Bull.* **63**, 1246-1253 (2018).
15. Geng, Z., *et al.* Achieving a record-high yield rate of 120.9 μg_{NH3} $mg_{cat.}^{-1}$ h^{-1} for N_2 electrochemical reduction over Ru single-atom Catalysts. *Adv. Mater.* **30**, 1803498 (2018).
16. Tao, H., *et al.* Nitrogen fixation by Ru single-atom electrocatalytic reduction. *Chem* **5**, 204-214 (2019).
17. Wang, M., *et al.* Over 56.55% faradaic efficiency of ambient ammonia synthesis enabled by positively shifting the reaction potential. *Nat. Commun.* **10**, 341 (2019).
18. Han, L., *et al.* Atomically dispersed molybdenum catalysts for efficient ambient nitrogen fixation. *Angew. Chem., Int. Ed.* **58**, 2321-2325 (2019).
19. Lü, F., *et al.* Nitrogen-coordinated single Fe sites for efficient electrocatalytic N_2 fixation in neutral media. *Nano Energy* **61**, 420-427 (2019).
20. Zhang, S., *et al.* Electrocatalytically Active Fe-$(O\text{-}C_2)_4$ single-atom sites for efficient reduction of nitrogen to ammonia. *Angew. Chem., Int. Ed.* **59**, 13423-13429 (2020).
21. He, C., *et al.* Identification of FeN_4 as an efficient active site for electrochemical N_2 Reduction. *ACS Catal.* 7311-7317 (2019).
22. Zhang, N., *et al.* Surface-regulated rhodium–antimony nanorods for nitrogen fixation. *Angew. Chem., Int. Ed.* **59**, 8066-8071 (2020).

23. Lv, C., *et al.* An amorphous noble-metal-free electrocatalyst that enables nitrogen fixation under ambient conditions. *Angew. Chem., Int. Ed.* **57**, 6073-6076 (2018).

24. Luo, Y., *et al.* Efficient electrocatalytic N_2 fixation with MXene under ambient conditions. *Joule* **3**, 279-289 (2019).

25. Fang, Y., *et al.* High-performance electrocatalytic conversion of N_2 to NH_3 using oxygen-vacancy-rich TiO_2 in situ grown on Ti_3C_2Tx MXene. *Adv. Energy Mater.* **0**, 1803406 (2019).

26. Qin, Q., *et al.* Enhanced electrocatalytic N_2 reduction via partial anion substitution in titanium oxide-carbon composites. *Angew. Chem., Int. Ed.* **58**, 13101-13106 (2019).

27. Cao, N., *et al.* Doping strain induced bi-Ti3$^+$ pairs for efficient N_2 activation and electrocatalytic fixation. *Nat. Commun.* **10**, 2877 (2019).

28. Zhang, G., Ji, Q. & Zhang, K. Triggering surface oxygen vacancies on atomic layered molybdenum dioxide for a low energy consumption path toward nitrogen fixation. *Nano Energy* **59**, 10-16 (2019).

29. Han, J., *et al.* Ambient N_2 fixation to NH_3 at ambient conditions: using Nb_2O_5 nanofiber as a high-performance electrocatalyst. *Nano Energy* **52**, 264-270 (2018).

30. Zhang, S., *et al.* Cu doping in CeO_2 to form multiple oxygen vacancies for dramatically enhanced ambient N_2 reduction performance. *Chem. Commun.* **55**, 2952-2955 (2019).

31. Wu, X., *et al.* Mn_3O_4 Nanocube: An efficient electrocatalyst toward artificial N_2 fixation to NH_3. *Small Methods* **14**, 1803111 (2018).

32. Hu, L., *et al.* Ambient electrochemical ammonia synthesis with high selectivity on Fe/Fe oxide Catalyst. *ACS Catal.* **8**, 9312-9319 (2018).

33. Yu, G., *et al.* Electrospun TiC/C nanofibers for ambient electrocatalytic N_2 reduction. *J. Mater. Chem. A* **7**, 19657-19661 (2019).

34. Zhang, L., *et al.* Electrochemical ammonia synthesis via nitrogen reduction reaction on a MoS_2 catalyst: theoretical and experimental studies. *Adv. Mater.* **30**, 1800191 (2018).

35. Guo, Y. et al. Boosting nitrogen reduction reaction by bio-inspired FeMoS containing hybrid electrocatalyst over a wide pH range. *Nano Energy* **62**, 282-288 (2019).

36. Chen, P., *et al.* Interfacial engineering of cobalt sulfide/graphene hybrids for highly efficient ammonia electrosynthesis. *Pnas* 201817881 (2019).

37. Li, P., *et al.* Ambient electrocatalytic N_2 reduction to NH_3 by metal fluorides. *J. Mater. Chem. A* **7**, 17761-17765 (2019).

38. Wang, H., *et al.* Direct fabrication of bi-metallic PdRu nanorod assemblies for electrochemical ammonia synthesis. *Nanoscale* **11**, 5499-5505 (2019).

39. Pang, F., *et al.* Bimodal nanoporous Pd_3Cu_1 alloy with restrained hydrogen evolution for stable and high yield electrochemical nitrogen reduction. *Nano Energy* **58**, 834-841 (2019).

40. Kumar, R.D., *et al.* Trimetallic PdCuIr with long-spined sea-urchin-like morphology for ambient electroreduction of nitrogen to ammonia. *J. Mater. Chem. A* **7**, 3190-3196 (2019).

41. Li, W., *et al.* Nitrogen-free commercial carbon cloth with rich defects for electrocatalytic ammonia synthesis under ambient conditions. *Chem. Commun.* **54**, 11188-11191 (2018).

42. Xia, L., *et al.* S-doped carbon nanospheres: An efficient electrocatalyst toward artificial N_2 fixation to NH_3. *Small Methods* **3**, 1800251 (2019).

43. Zhao, J., *et al.* Defect-rich fluorographene nanosheets for artificial N_2 fixation under ambient conditions. *Chem. Commun.* **55**, 4266-4269 (2019).

44. Zhang, L., Ding, L.X., Chen, G. F., Yang, X. & Wang, H. Ammonia synthesis under ambient conditions: selective electroreduction of dinitrogen to ammonia on black phosphorus nanosheets. *Angew. Chem., Int. Ed.* **58**, 2612-2616 (2019).

45. Yu, X., *et al.* Boron-doped graphene for electrocatalytic N_2 reduction. *Joule* **2**, 1610-1622 (2018).

46. Chen, C., *et al.* B N pairs enriched defective carbon nanosheets for ammonia synthesis with high efficiency. *Small Methods* **15**, 1805029 (2019).

47. Liu, Y., *et al.* Facile ammonia synthesis from electrocatalytic N_2 reduction under ambient conditions on N-doped porous carbon. *ACS Catal.* **8**, 1186-1191 (2018).

48. Yang, X., *et al.* Mechanistic Insights into electrochemical nitrogen reduction reaction on vanadium Nitride nanoparticles. *J. Am. Chem. Soc.* **140**, 13387-13391 (2018).

49. Zhao, J., *et al.* High-performance N_2-to-NH_3 fixation by a metal-free electrocatalyst. *Nanoscale* **11**, 4231-4235 (2019).

50. Lv, C., *et al.* Defect engineering metal-free polymeric carbon nitride electrocatalyst for effective nitrogen fixation under ambient conditions. *Angew. Chem., Int. Ed.* **57**, 10246-10250 (2018).

51. Qiu, W., *et al.* High-performance artificial nitrogen fixation at ambient conditions using a metal-free electrocatalyst. *Nat. Commun.* **9**, 3485 (2018).

52. Andersen, S.Z., *et al.* A rigorous electrochemical ammonia synthesis protocol with quantitative isotope measurements. *Nature* **570**, 504-508 (2019).

53. Ma, L., *et al.* In situ DRIFTS and temperature-programmed technology study on NH_3-SCR of NO_x over Cu-SSZ-13 and Cu-SAPO-34 catalysts. *Appl. Catal. B Environ.* **156-157**, 428-437 (2014).

54. Ma, L., *et al.* Characterization of commercial Cu-SSZ-13 and Cu-SAPO-34 catalysts with hydrothermal treatment for NH_3-SCR of NO_x in diesel exhaust. *Chem. Eng. J.* **225**, 323-330 (2013).

55. Ye, Q., Wang, L. & Yang, R.T. Activity, propene poisoning resistance and hydrothermal stability of copper exchanged chabazite-like zeolite catalysts for SCR of NO with ammonia in comparison to Cu/ZSM-5. *Appl. Catal. A Gen.* **427-428**, 24-34 (2012).
